# Adverse pregnancy outcomes in women with type 1 diabetes are associated with multiple alterations in the vaginal microbiome

**DOI:** 10.1007/s00125-025-06509-0

**Published:** 2025-08-07

**Authors:** Alexandra J. Roth-Schulze, Esther Bandala-Sanchez, Katrina M. Ngui, Gaetano Naselli, Helena Oakey, Patricia Ashwood, Guinevere Martin, James D. Brown, Enrique Zozaya-Valdés, Rebecca L. Thomson, Peter G. Colman, John M. Wentworth, Peter J. Vuillermin, Tony Hunyh, Georgia Soldatos, Jennifer J. Couper, Megan A. S. Penno, Leonard C. Harrison

**Affiliations:** 1 Walter and Eliza Hall Institute of Medical Research, Melbourne, VIC Australia; 2 Department of Medical Biology, University of Melbourne, Melbourne, VIC Australia; 3 Robinson Research Institute, Adelaide Medical School, University of Adelaide, Adelaide, SA Australia; 4 Department of Diabetes and Endocrinology, Royal Melbourne Hospital, Melbourne, VIC Australia; 5 Faculty of School of Medicine, Deakin University, Geelong, VIC Australia; 6 Child Health Research Unit, Barwon Health, Geelong, VIC Australia; 7 Children’s Health Research Centre, Faculty of Medicine, The University of Queensland, South Brisbane, QLD Australia; 8 Department of Endocrinology and Diabetes, Queensland Children’s Hospital, South Brisbane, QLD Australia; 9 Monash Centre for Health Research and Implementation, School of Public Health and Preventive Medicine, Monash University, Melbourne, VIC Australia; 10 Diabetes and Vascular Medicine Unit, Monash Health, Melbourne, VIC Australia

**Keywords:** *Candida*, Community state types, *Gardnerella vaginalis*, Internal transcribed spacer 1, ITS1, Lactobacilli, *Malassezia*, Microbiome, Mycobiome, Pre-eclampsia, Pregnancy, Pre-term birth, 16S rRNA gene, T1D, Type 1 diabetes

## Abstract

**Aims/hypothesis:**

The vaginal microbiome has been linked to adverse pregnancy outcomes, which are markedly increased in women with type 1 diabetes. To investigate this relationship, we profiled the vaginal microbiome in pregnant women with and without type 1 diabetes, and in relation to pre-term birth (PTB) and pre-eclampsia (PE) in women with type 1 diabetes.

**Methods:**

Bacterial and fungal microbiomes were analysed by 16S rRNA gene and internal transcribed spacer 1 sequencing, respectively, in the third trimester of 310 pregnancies (160 with type 1 diabetes) for bacteria, and 147 pregnancies (70 with type 1 diabetes) for fungi.

**Results:**

The vaginal microbiome was altered by type 1 diabetes in pregnancy, with an increase in the bacterial species *Lactobacillus iners* and *Lactobacillus jensenii*, and in the anaerobic genera *Gardnerella*, *Anaerococcus*, *Prevotella*, *Dialister*, *Peptoniphilus* and others that are associated with vaginal dysbiosis. In addition, the abundance of the fungal species *Malassezia restricta* was increased in women with type 1 diabetes. These changes were associated with increased risks of PTB and PE. PTB was associated with higher bacterial alpha diversity, decreased abundance of *Lactobacillus reuteri*, and increased abundance of *Malassezia* fungal genus, family Malasseziaceae and order Malasseziales. PE was associated with higher bacterial alpha diversity, increased abundance of *Gardnerella vaginalis* and decreased abundance of *Candida albicans*.

**Conclusions/interpretation:**

Adverse pregnancy outcomes in women with type 1 diabetes are reflected by distinct changes in the vaginal microbiome. This highlights the importance of monitoring and managing the vaginal microbiome in high-risk pregnancies, particularly those complicated by type 1 diabetes. Early detection and treatment of risk-associated taxa, e.g. *G. vaginalis* in the case of PE, could potentially improve vaginal health and pregnancy outcomes in women with type 1 diabetes.

**Graphical Abstract:**

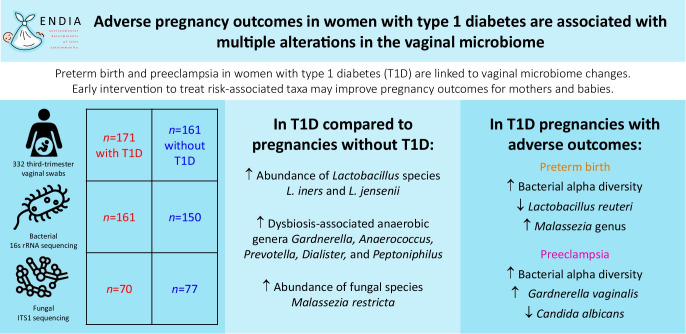

**Supplementary Information:**

The online version of this article  (10.1007/s00125-025-06509-0) contains peer-reviewed but unedited supplementary material.


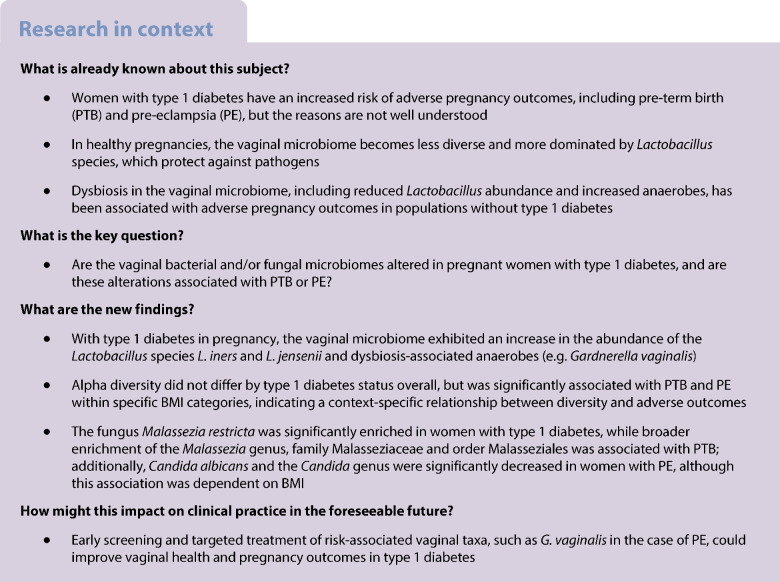


## Introduction

The vaginal microbiome in healthy women is well characterised. Lactobacilli predominate and are classified into five community state types (CSTs), four of which are dominated by a single *Lactobacillus* species [1]. In healthy pregnancies, the vaginal bacterial microbiome becomes more stable and less diverse, as the abundance of lactobacilli increases [2–4]. In the few studies in which the vaginal microbiome has been studied in pregnancies with adverse outcomes, bacterial diversity was increased and the abundance of *Lactobacillus* species decreased [4–6]. Vaginal fungi are also altered in pregnancy, notably an increase in *Candida* species, usually *Candida albicans* [7]. Pregnancy in women with type 1 diabetes is associated with a much higher risk of adverse outcomes, including pre-term birth (PTB) (OR 2.88) and pre-eclampsia (PE) (OR 4.2) [8, 9]. However, the vaginal microbiome has not been characterised in women with type 1 diabetes in pregnancy. Type 1 diabetes may shape the vaginal microbiome in several compounding ways. Raised concentrations of extracellular glucose could promote the growth of vaginal microorganisms, as in the case of *Candida* [7], and of bacteria such as *Gardnerella vaginalis*, which is implicated in bacterial vaginosis [10]. Impaired immune function in type 1 diabetes [11] could be associated with compositional changes in the microbiome and the emergence of abnormal species. In addition, hormonal changes in women with poorly controlled type 1 diabetes may alter the vaginal microbiome. For example, oestrogen plays a crucial role in maintaining the health of the vagina by promoting glycogen production in epithelial cells. Glycogen is metabolised by lactobacilli to lactic acid, to maintain an acidic vaginal pH, which inhibits the growth of other bacteria [12].

To characterise the vaginal microbiome in pregnant women with and without type 1 diabetes, and its relationship to PTB and PE in women with type 1 diabetes, we analysed the vaginal bacterial microbiome by 16S rRNA gene sequencing and the fungal microbiome by internal transcribed spacer 1 (ITS1) sequencing.

## Methods

### Study population

**Fig. 1:**
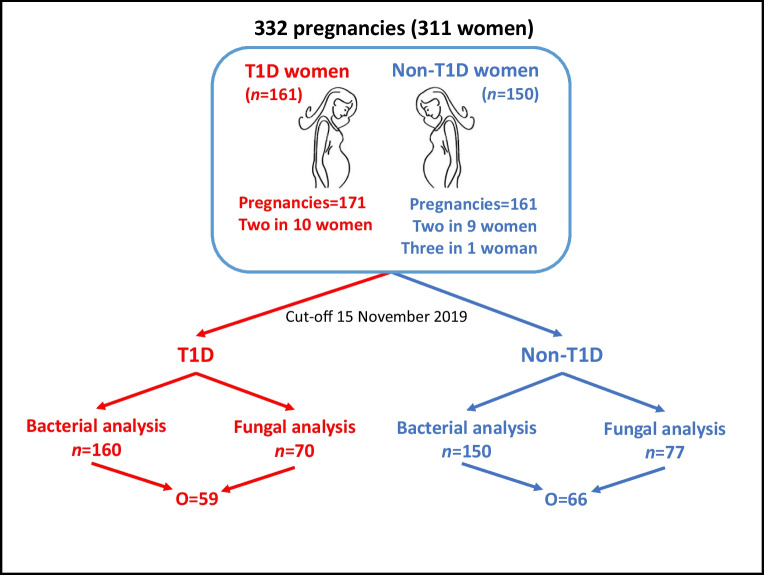
Numbers of vaginal swab samples obtained in the third trimester of pregnancy in women with type 1 diabetes (T1D) and women without type 1 diabetes (non-T1D). *n*, number of samples; O, number of overlapping samples between bacterial and fungal microbiome analyses

Using a cut-off date of 15 November 2019, we collected all available third-trimester (T3) vaginal swab samples from pregnant women with type 1 diabetes (*n*=161) and those without type 1 diabetes (*n*=150), enrolled in the Environmental Determinants of Islet Autoimmunity (ENDIA) pregnancy–birth cohort study [13]. The mean gestational age at sampling (± SD) was 34.24 ± 2.57 weeks. Ten women with type 1 diabetes and nine without type 1 diabetes provided samples for two different pregnancies, and one without type 1 diabetes provided samples for three pregnancies, resulting in 332 samples (Fig. 1). No repeat contributors had prior PTB or PE. The main criterion for participation in ENDIA was a child with a first-degree relative with type 1 diabetes. Women who provided written informed consent were enrolled in the study between 2013 and 2019 at eight clinical sites in Australia. The study was approved nationally by the Women’s and Children’s Health Network Human Research Ethics Committee in Adelaide, acting as the lead under the Australian National Mutual Acceptance Scheme (reference number 2020/HRE01400). Conduct in Western Australia is approved by the Child and Adolescent Health Service Human Research Ethics Committee (RGS0000002402). Governance approval was obtained at all participating sites. The investigators and study staff performed the study in accordance with International Conference on Harmonisation Good Clinical Practice guidelines, the Declaration of Helsinki, the Australian National Statement on Ethical Conduct in Human Research, and the Australian Code for the Responsible Conduct of Research. All participants provided written informed consent and were free to withdraw from the study at any time. Women self-reported their ethnicity. Demographic, pregnancy and birth data were collected prospectively, and clinical data were retrospectively verified against medical records.

### Vaginal sample collection and DNA extraction

The women participating in ENDIA self-collected vaginal samples using a Copan eSwab (Copan Italia SpA) early in the third trimester of pregnancy. DNA was extracted from the swab and medium using a Maxwell RSC Purefood and GMO Kit (Promega). DNA concentration was determined fluorometrically using an Invitrogen Qubit dsDNA Assay kit (ThermoFisher Scientific), and purity was assessed based on 260/280 nm and 260/230 nm absorbance ratios, as determined using a DeNovix DS-11 Spectrophotometer (DeNovix Inc.). DNA integrity was then checked by agarose gel electrophoresis. Samples were stored at −20°C until sequencing.

### Bacterial and fungal DNA marker amplification and sequencing

The V4 region of the 16S rRNA gene was amplified using primers 515F/806R for bacterial analysis [14] and the ITS1 region with ITS1F/ITS1R primers for fungal analysis [15]. Bacterial and fungal PCR products were visualised by agarose gel electrophoresis and quantified using Agilent 4200 TapeStation System. Equimolar amounts of purified amplicons were pooled and sequenced on the Illumina MiSeq platform using the MiSeq version 3 600 cycle (2 × 300 bp) kit.

### Bacterial sequence and CST cluster analysis

16S rRNA gene amplicon data from two sequencing runs were processed separately using QIIME2 (version 2024.10) [16]. Features (amplicon sequence variants; ASVs) were generated using ‘qiime dada2 denoise-paired’ [17]. Feature tables and representative sequences from both runs were merged, and taxonomy was assigned using a naive Bayes classifier pre-trained on the SILVA 16S database (version 138, 99% similarity) [18]. A phylogenetic tree was constructed within QIIME2 using MAFFT for alignment and FastTree for approximate maximum-likelihood inference. Representative features that could not be classified at the species level were improved by sequence alignment using BLASTn against the NCBI 16S ribosomal RNA database [19] (accessed January 2025) (see electronic supplementary material [ESM] Methods: BLAST features classification and ESM Table 1). The taxonomic profile was imported into the ‘phyloseq’ package [20] v1.50.0 in R (version 4.4.2 2024-10-31) [21]. ASVs were agglomerated into operational taxonomic units (OTUs) using the phylogenetic tree (with a co-phenetic distance of 0.03 using tip_glom). OTUs contributing <0.01% of total abundance were removed. OTUs sharing the same species classification were merged. To standardise sequencing depth (ranging from approximately 10,000 to 100,000 reads), all samples were rarefied to 7000 sequences without replacement using rarefy_even_depth. This threshold balanced sample retention with adequate depth to capture variation in alpha and beta diversity. Normalised data were used for all statistical analyses, except for differential abundance analysis.

For CST frequency analysis, samples were clustered into six groups based on dominant OTU classification (see ESM Methods: Analysis of vaginal CSTs).

### Fungal sequence analysis

ITS1 amplicon data were processed using QIIME2, using only single-end reads (read 1) to avoid data loss from the variable ITS1 region length (300–900 bp). Representative sequences from read 1 were taxonomically classified using a naive Bayes classifier trained on the UNITE version 10 (2024) ITS database [22], and a phylogenetic tree was built as described for 16S data. The resulting taxonomic profile (ASV counts per sample) was imported into ‘phyloseq’. ASVs contributing <0.01% of total abundance and those unclassified at the kingdom level were excluded. ASVs were agglomerated into species hypotheses (SH), defined in UNITE as clusters with ≥99% identity representing species or species-like units. The SH system offers a consistent, sequence-based classification framework for fungal diversity. All samples were rarefied to 5000 sequences for normalisation, and normalised data were used for all statistical analyses, except for differential abundance analysis.

### HLA typing

HLA class II DR alleles were imputed by typing selected SNPs validated by machine learning to predict HLA risk alleles for type 1 diabetes, as previously reported [23]. For analysis, HLA types were classified as DR3,4 (high type 1 diabetes risk ), any combination of DR3 and DR4 other than 3,4, i.e. DR3,X, DR4,X, DR3,3 or DR4,4 (intermediate type 1 diabetes risk), and DRX,X (low type 1 diabetes risk).

### Alpha diversity analysis

Alpha diversity (diversity within microbial communities), determined as richness (total number of OTUs), Chao1 index, Shannon index and the InvSimpson index, were calculated using the function ‘estimate_richness’ from ‘phyloseq’. Statistical differences in bacterial and fungal alpha diversity between groups of interest were tested by fitting generalised linear mixed models using the glmmTMB package [24] v1.1.10 (ESM Methods: Alpha diversity).

### Beta diversity analysis

Beta diversity (diversity between microbial communities) was determined using ‘phyloseq’ (function distance, method = ‘bray’) on proportional untransformed and log-transformed [i.e. base=exp(1)] data [i.e. log (*x *+ 1)]. This function calculates Bray–Curtis coefficients, measuring the dissimilarity between communities based on their taxa and taxa abundances. Statistical differences in beta diversity were evaluated using a modified version of the Adonis function from the ‘vegan’ package [25] v2.6-8, which performs a repeated measure-aware permutation ANOVA (ESM Methods: Beta diversity).

### Taxa composition analysis

To identify differences in specific taxa between women with and without type 1 diabetes, who gave birth at term or pre-term, or did and did not develop PE, differential abundance was analysed in limma for the bacterial microbiome and by fitting glmmTMB models for the mycobiome (ESM Methods: Taxa composition analysis).

## Results

Vaginal swab samples were available from 332 pregnancies from 311 women with one or more pregnancies (Fig. 1). Their characteristics are detailed in Tables 1 and 2 and ESM Table 2. For analysis of the bacterial microbiome, 22 of the 332 samples were removed because they yielded fewer than 7000 sequences, leaving 310. For analysis of the fungal microbiome, 52 of the 332 samples were removed because no sequence was obtained, four were removed because they yielded fewer than 5000 sequences, and 129 were not sequenced because insufficient DNA remained after bacterial analysis, leaving 147. Bacterial and fungal sequences were both analysed on 125 samples. Non-overlapping bacterial and fungal samples numbered 185 and 22, respectively. Of the 310 DNA samples analysed by 16S rRNA gene sequencing, 160 were from women with type 1 diabetes and 150 were from women without type 1 diabetes. Of the 147 DNA samples analysed by ITS-amplicon sequencing, 70 were from women with type 1 diabetes and 77 were from women without type 1 diabetes (Fig. 1).

**Table 1:** Summary of maternal characteristics for non-type 1 diabetes pregnancies and type 1 diabetes pregnancies (bacterial microbiome)

	T1D status		PTB		PE	
Characteristic	Non-T1D (*N*=150)	T1D (*N*=160)	Term (*N*=104)	Premature (*N*=44)^a^	No PE (*N*=129)	PE (*N*=29)
Age at conception (years)	32.2 ± 4.5	31.9 ± 4.7	32.0 ± 4.2	31.6 ± 5.7	32.1 ± 4.3	30.8 ± 6.3
Birth country						
Australia	121 (80.7)	126 (78.8)	83 (79.8)	34 (77.2)	101 (78.2)	23 (79.3)
Overseas	25 (16.7)	31 (19.4)	19 (18.3)	9 (20.5)	26 (20.2)	5 (17.2)
Missing	4 (2.7)	3 (1.9)	2 (1.9)	1 (2.3)	2 (1.6)	1 (3.4)
HLA-DR						
DR34	12 (8.0)	51 (31.9)	33 (31.7)	13 (29.6)	42 (32.6)	8 (27.6)
DR4X, DR44	49 (32.7)	62 (38.8)	41 (39.4)	17 (38.6)	51 (39.5)	11 (37.9)
DR3X, DR33	34 (22.7)	30 (18.8)	20 (19.2)	7 (15.9)	23 (17.8)	6 (20.7)
DRXX	54 (36.0)	16 (10.0)	10 (9.6)	6 (13.6)	13 (10.1)	3 (10.3)
Indeterminate	1 (0.7)	1 (0.6)	0 (0.0)	1 (2.3)	0 (0.0)	1 (3.5)
Mode of birth						
Caesarean (emergency)	24 (16.0)	58 (36.3)	23 (22.1)	35 (79.6)	35 (27.1)	23 (79.3)
Premature caesarean (elective)	1 (0.7)	12 (7.5)	0 (0.0)	0 (0.0)^a^	10 (7.80)	2 (6.9)
Term caesarean (elective)	22 (14.7)	45 (28.1)	45 (43.3)	0 (0.0)	45 (34.9)	0 (0.0)
Vaginal (instrumental)	23 (15.3)	18 (11.3)	13 (12.5)	5 (11.4)	15 (11.6)	2 (6.9)
Vaginal (non-instrumental)	80 (53.3)	27 (16.9)	23 (22.1)	4 (9.1)	24 (18.6)	2 (6.9)
Pre-pregnancy BMI (kg/m^2^)	23.7 (21.4–28.0)	25.6 (22.3–30.6)	24.2 (21.7–29.9)	28 (25.6–32.2)	24.6 (22.0–30.2)	28.5 (25.6–33.0)
PE	0 (0.00)	29 (18.13)	6 (5.77)	21 (47.73)		
Missing	9 (6.0)	2 (1.25)	2 (1.9)	0 (0)		
GA at birth (weeks)	39.3 ± 1.4	37.1 ± 1.4	37.9 ± 0.6	35.5 ± 1.2	37.4 ± 1.2	35.8 ± 1.3
Parity						
0	68 (45.3)	85 (53.1)	50 (48.1)	31 (70.5)	64 (49.6)	20 (69.0)
1	48 (32.0)	54 (33.8)	40 (38.5)	9 (20.5)	47 (36.4)	7 (24.1)
>1	34 (22.7)	21 (13.1)	14 (13.5)	4 (9.1)	18 (14.0)	2 (6.9)
HbA_1c_ (mmol/mol)	NA	45 (40–53)	44 (39–50)	49 (43–57)	44 (40–50)	56 (48–61)
HbA_1c_ (%)	NA	6.25 (5.8–7.0)	6.15 (5.7–6.7)	6.6 (6.1–7.4)	6.2 (5.8–6.7)	7.3 (6.5–7.7)
Missing	NA	14 (8.8)	10 (9.6)	3 (6.8)	10 (7.75)	2 (6.9)
PTB	7 (4.7)	44 (29.7)^a^			33 (25.6)	23 (79.3)

Values for continuous variables are means ± SD or medians (IQR); values for categorical variables are *n* (%)

Unless otherwise stated, percentages were calculated including missing data in the denominator

^a^Excludes 12 elective caesareans at <37 weeks’ gestation

GA, gestational age; T1D, type 1 diabetes

**Table 2:** Summary of maternal characteristics for non-type 1 diabetes pregnancies and type 1 diabetes pregnancies (fungal microbiome)

	T1D status		PTB		PE	
Characteristic	Non-T1D (*N*=77)	T1D (*N*=70)	Term (*N*=45)	Premature (*N*=17)^a^	No PE (*N*=57)	PE (*N*=11)
Age at conception (years)	31.8 ± 5.0	32.3 ± 4.8	33.3 ± 4.0	31.9 ± 6.0	32.8 ± 4.3	30.1 ± 6.5
Birth country						
Australia	59 (76.6)	51 (72.9)	33 (73.3)	12 (70.6)	42 (73.7)	7 (63.6)
Overseas	15 (19.5)	17 (24.3)	11 (24.4)	4 (23.5)	14 (24.6)	3 (27.3)
Missing	3 (3.9)	2 (2.9)	1 (2.2)	1 (5.9)	1 (1.8)	1 (9.1)
HLA-DR						
DR34	5 (6.5)	24 (34.3)	16 (35.6)	6 (35.3)	19 (33.3)	4 (36.4)
DR4X, DR44	20 (26.0)	24 (34.3)	14 (31.1)	6 (35.3)	20 (35.1)	4 (36.4)
DR3X, DR33	19 (24.7)	13 (18.6)	10 (22.2)	1 (5.9)	10 (17.5)	2 (18.2)
DRXX	33 (42.9)	9 (12.9)	5 (11.1)	4 (23.5)	8 (14.0)	1 (9.1)
Indeterminate	0 (0.0)	0 (0.0)	0 (0.0)	0 (0.0)	0 (0.0)	0 (0.0)
Mode of birth						
Caesarean (emergency)	12 (15.6)	24 (34.3)	10 (22.2)	14 (82.4)	16 (28.1)	8 (72.7)
Premature caesarean (elective)	0 (0.0)	8 (11.4)	0 (0.0)	0 (0.0)^a^	6 (10.5)	2 (18.2)
Term caesarean (elective)	15 (19.5)	20 (28.6)	20 (44.4)	0 (0.0)	20 (35.1)	0 (0.0)
Vaginal (instrumental)	10 (13.0)	5 (7.1)	4 (8.9)	1 (5.9)	4 (7.0)	0 (0.0)
Vaginal (non-instrumental)	40 (52.0)	13 (18.6)	11 (24.4)	2 (11.8)	11 (19.3)	1 (9.1)
Pre-pregnancy BMI (kg/m^2^)	23.3 (21.1–27.0)	25.5 (22.5–30.5)	24.3 (22.2–30.5)	27.4 (24.4–30.1)	24.44 (22.3–30.4)	29.11 (25.8–33.5)
Missing	2 (2.6)	3 (4.3)	1 (2.2)	2 (11.8)	2 (3.5)	1 ( 9.1)
PE	0 (0.0)	11 (15.7)	2 (4.5)	7 (41.2)		
Missing	2 (2.7)	2 (2.9)	2 (4.5)	0 (0.0)		
GA at birth (weeks)	39.5 (1.3)	37.0 (1.4)	37.9 (0.6)	35.24 (1.3)	37.3 (1.3)	35.61 (1.5)
Parity						
0	37 (48.0)	32 (45.7)	19 (42.2)	12 (70.59)	24 (42.1)	7 (63.6)
1	24 (31.2)	27 (38.6)	19 (42.2)	3 (17.65)	25 (43.9)	2 (18.2)
>1	16 (20.8)	11 (15.7)	7 (15.6)	2 (11.76)	8 (14.0)	2 (18.2)
HbA_1c_ (mmol/mol)	NA	48 (41–55)	44 (40–52)	43 (38–50)	50 (44–57)	58 (53–64)
HbA_1c_ (%)	NA	6.50 (5.9–7.2)	6.2 (5.8–6.9)	6.1 (5.6–6.7)	6.7 (6.2–7.4)	7.5 (7–8)
Missing	NA	6 (8.6)	5 (11)	1 (5.9)	4 (7)	0 (0)
PTB	2 (2.6)	17 (24.3)^a^			16 (28.1)	9 (81.8)

Values for continuous variables are means ± SD or medians (IQR); values for categorical variables are *n* (%)

^a^Excludes eight elective caesareans at <37 weeks’ gestation

GA, gestational age; T1D, type 1 diabetes

After quality filtering, 57,334 ± 20,869 paired-end bacterial reads and 24,451 ± 14,206 paired-end fungal reads per sample (means ± SD) were obtained by Illumina MiSeq sequencing. Overall, a total of 90 bacterial species and 278 fungal OTUs were identified, with means (± SD) of 16.4 ± 10.6 and 8.2 ± 5.9 per sample, respectively.

### Changes in vaginal bacteria in pregnancy in women with type 1 diabetes

The 90 bacterial species were classified into six phyla: 79.7% Firmicutes, 14.4% Actinobacteriota, 5.4% Bacteroidota, 0.24% Campylobacterota, 0.14% Fusobacteriota and 0.11% Proteobacteria. *Lactobacillus crispatus* and *Lactobacillus iners* (Firmicutes) dominated the microbiome, comprising 38% and 24% of sequences, respectively, followed by *G. vaginalis* (Actinobacteria), *Lactobacillus gasseri* and *Lactobacillus jensenii* (ESM Fig. 1).

The vaginal bacterial microbiome was classified into six CSTs, four of which were dominated by *Lactobacillus* species [1] (ESM Results: Community state types). After adjusting for parity and BMI, CST distributions and *Lactobacillus* frequencies were similar between women with and without type 1 diabetes (ESM Tables 3 and 4 and ESM Fig. 2).

Alpha and beta diversity were also similar across type 1 diabetes status, with no interactions by BMI or parity, except for an interaction between type 1 diabetes and BMI for beta diversity using untransformed data. However, within BMI categories, type 1 diabetes-related differences were not significant (Table 3, ESM Tables 5 and 6, and ESM Figs 4 and 5). In the beta diversity analysis, interactions between type 1 diabetes status and HLA class II type were detected at all taxonomic levels above species. HLA-associated differences were found in women with and without type 1 diabetes when using log-transformed Bray–Curtis distances; however, when untransformed data were used, these differences were restricted to women with type 1 diabetes (ESM Table 6). Significant HLA-related differences by type 1 diabetes status were detected for intermediate type 1 diabetes risk HLA-DR types (HLA-DR3,X, HLA-DR4,X, HLA-DR3,3 and HLA-DR4,4 combined) and low-risk HLA-DRX,X, but not for high-risk HLA-DR3,4 across both untransformed and log-transformed beta diversity data (ESM Table 6).

**Table 3:** Vaginal bacterial and fungal microbiome analysis: *p* values according to type 1 diabetes and adverse pregnancy outcome status

	T1D status	PTB	PE
Bacterial			
Alpha diversity (adjusted *p* value)			
Richness	0.92	0.94	0.65
Chao1	0.92	0.94	0.65
InvSimpson	0.92	0.01**^a^; 0.74^b^; 0.11^c^	0.024*^a^; 0.07^b^; 0.17^c^
Shannon	0.92	0.94	0.65
Beta diversity (adjusted *p* value)			
Species	0.39	0.54	0.30
Genus	0.39	0.54	0.30
Family	0.39	0.54	0.30
Order	0.39	0.54	0.30
Phylum	0.39	0.54	0.30
Fungal			
Alpha diversity			
Richness	0.79	0.37	1.00
Chao1	0.79	0.37	1.00
InvSimpson	0.79	0.37	1.00
Shannon	0.79	0.56	1.00
Beta diversity			
Species	0.07	0.75	0.71
Genus	0.07	0.75	0.71
Family	0.07	0.75	0.71
Order	0.58	0.75	0.71
Phylum	0.58	0.82	0.71

Type 1 diabetes and pregnancy complication status are included in the analysis as exposures, with interactions between these exposures and confounders BMI and parity. A separate model was fitted for each exposure (type 1 diabetes, PTB and PE) and each response

Adjusted *p* values represent main effects of exposures unless otherwise stated (i.e. an interaction with the confounder was present). The beta diversity adjusted *p* values were obtained from tests performed on log-transformed data

^*^p<0.05, **p<0.01

^a^BMI category of normal-weight women; ^b^BMI category of overweight women; ^c^BMI category of women with obesity

**Fig. 2:**
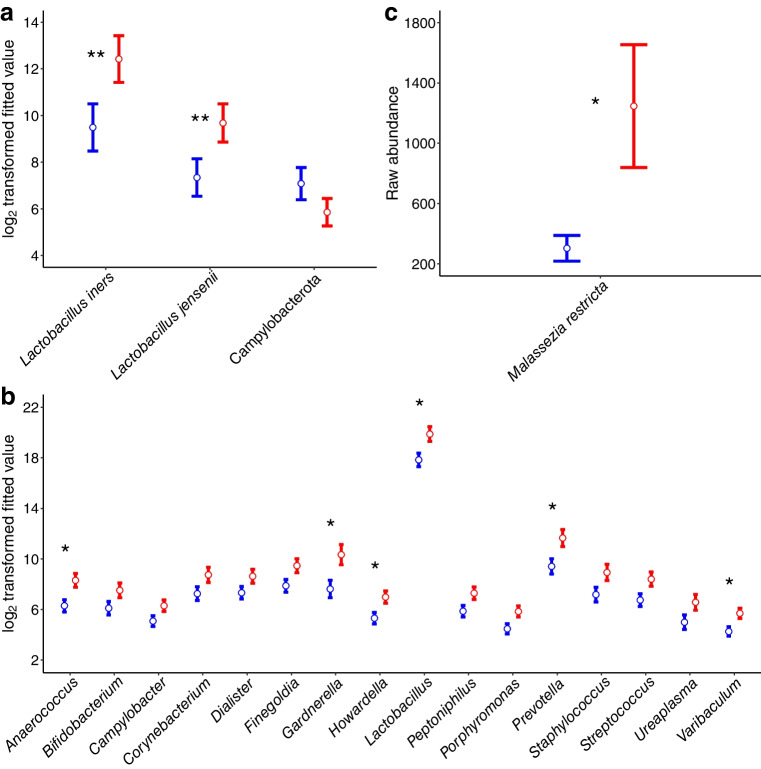
Differences in the vaginal bacterial microbiome and mycobiome between women with type 1 diabetes (red) and those without (blue). (**a**) Differentially abundant bacterial taxa by type 1 diabetes status. Values are means with SEM. (**b**) Differentially abundant bacterial genera by type 1 diabetes status in normal-weight women. Values are means with SEM. (**c**) Differentially abundant fungi. Values are means with SEM. *p<0.05, **p<0.01, between women with type 1 diabetes and those without

Differential abundance analysis showed an increased abundance of *L. jensenii* and *L. iners* and a decrease in Campylobacterota in women with type 1 diabetes (Fig. 2a, Table 4 and ESM Table 7). The Campylobacterota comprised the species *Campylobacter hominis* and *Campylobacter ureolyticus*, with the latter being dominant. Sixteen genera, including anaerobic bacteria of the genera *Gardnerella*, *Prevotella*, *Anaerococcus*, *Varibaculum*, *Porphyromonas*, *Peptoniphilus*, *Dialister*, *Finegoldia*, *Ureaplasma* and *Corynebacterium*, were enriched in women with type 1 diabetes, but only among the normal-weight group (Fig. 2b). All *Gardnerella* reads were assigned to *G. vaginalis*, indicating no other *Gardnerella* species were detected. Because significant differences in beta diversity were detected due to HLA type, we also tested differential abundance by HLA type, to exclude the possibility that HLA was driving differences attributed to type 1 diabetes status. No differences were found by HLA alone. Within intermediate and low-risk HLA-DR types but not the high-risk HLA-DR3,4, differences by type 1 diabetes status were detected similarly to the beta diversity analysis (ESM Table 7).

**Table 4:** Differentially abundant bacterial and fungal taxa according to type 1 diabetes and adverse pregnancy outcome status

Comparison	Group	Taxa	Enriched	logFC/estimate^a^
Bacterial				
T1D vs non-T1D	All women	Species: *Lactobacillus jensenii*	T1D	−2.3
T1D vs non-T1D	All women	Species: *Lactobacillus iners*	T1D	−2.9
T1D vs non-T1D	All women	Phylum: Campylobacterota	Non-T1D	1.2
T1D vs non-T1D	Normal-weight women	Genus: *Anaerococcus*	T1D	−2.0
T1D vs non-T1D	Normal-weight women	Genus: *Varibaculum*	T1D	−1.4
T1D vs non-T1D	Normal-weight women	Genus: *Lactobacillus*	T1D	−2.0
T1D vs non-T1D	Normal-weight women	Genus: *Gardnerella*	T1D	−2.7
T1D vs non-T1D	Normal-weight women	Genus: *Howardella*	T1D	−1.7
T1D vs non-T1D	Normal-weight women	Genus: *Prevotella*	T1D	−2.2
T1D vs non-T1D	Normal-weight women	Genus: *Porphyromona*	T1D	−1.4
T1D vs non-T1D	Normal-weight women	Genus:* Streptococcus*	T1D	−1.7
T1D vs non-T1D	Normal-weight women	Genus: *Peptoniphilus*	T1D	−1.4
T1D vs non-T1D	Normal-weight women	Genus: *Finegoldia*	T1D	−1.6
T1D vs non-T1D	Normal-weight women	Genus:* Staphylococcus*	T1D	−1.7
T1D vs non-T1D	Normal-weight women	Genus:* Campylobacter*	T1D	−1.2
T1D vs non-T1D	Normal-weight women	Genus:* Corynebacterium*	T1D	−1.5
T1D vs non-T1D	Normal-weight women	Genus: *Ureaplasma*	T1D	−1.6
T1D vs non-T1D	Normal-weight women	Genus:* Bifidobacterium*	T1D	−1.4
T1D vs non-T1D	Normal-weight women	Genus: *Dialister*	T1D	−1.3
PTB vs non-PTB	Women with T1D	Species: *Lactobacillus reuteri*	Non-PTB	−2.0
PE vs non-PE	Women with T1D	Species: *Gardnerella vaginalis*	PE	−5.0
Fungal				
T1D vs non-T1D	All women	Species: *Malassezia restricta*	T1D	1.4
PTB vs non-PTB	Women with T1D	Genus: *Malassezia*	PTB	−2.0
PTB vs non-PTB	Women with T1D	Family: Malasseziaceae	PTB	−2.0
PTB vs non-PTB	Women with T1D	Order: Malasseziales	PTB	−2.4
PE vs non-PE	Normal-weight women with T1D	Species: *Candida albicans*	Non-PE	0.2
PE vs non-PE	Normal-weight women with T1D	Genus: *Candida*	Non-PE	2.4
PE vs non-PE	Obese women with T1D		PE	−1.4
PE vs non-PE	Women with T1D	Order: Polyporales	Non-PE	−3.4

^a^Values for the bacterial comparisons are logFC, log_2_ fold change; those for the fungal comparisons are estimates

logFC, natural log scale (i.e. log base e) fold change

### Changes in vaginal fungi in pregnancy in women with type 1 diabetes

Of the 278 fungal species or SH, 267 (96%) were annotated up to the phylum level. Of the remaining species, ten were classified as phylum ‘Fungi_phy_Incertae_sedis’ (i.e. fungal sequences that cannot be confidently assigned to any known phylum) and one was classified only as fungal (kingdom level). The two phyla detected, Basidiomycota and Ascomycota, accounted for 59% and 41% of the classified SHs and 39% and 61% of the classified sequences, respectively. One SH classified to the species *Candida albicans* (phylum Ascomycota) dominated the mycobiome community, representing 37% of all fungal sequences on average per sample, followed by *Nakaseomyces* spp. (3.2%) and *Malassezia restricta* (3.1%) (ESM Fig. 3).

No interactions or significant differences due to type 1 diabetes status were detected for alpha diversity (ESM Table 5). Differences in beta diversity related to type 1 diabetes status were observed only when using log-transformed data, but these associations did not remain statistically significant after correction for multiple testing. Nonetheless, the pattern suggests that low-abundance taxa may contribute to community-level differences, as their influence is enhanced in log-transformed analyses and diminished in untransformed data (Table 3, ESM Table 6). No differences due to HLA were found (ESM Table 6). Generalised linear mixed models (glmmTMB) were fitted separately for taxa with a prevalence greater than 25% across samples. For each taxon, the best-fitting model was selected based on model fit criteria. This approach identified a significant enrichment of *M. restricta* in women with type 1 diabetes (Fig. 2c, Table 4 and ESM Table 7).

### The vaginal microbiome and adverse pregnancy outcomes

Vaginal microbiome diversity and composition were examined in relation to PTB and PE.

#### Pre-term birth

**Fig. 3:**
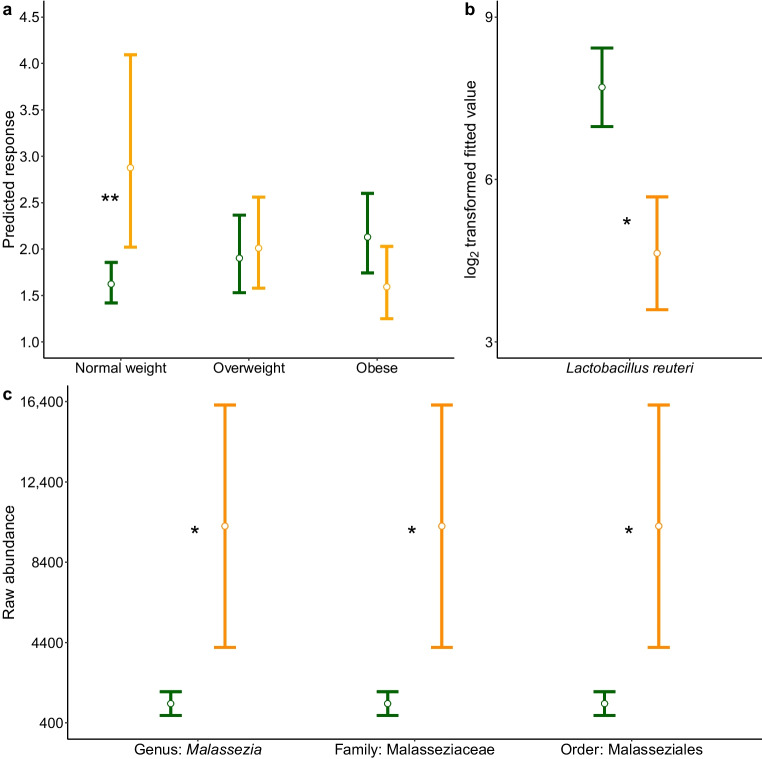
Differences in the vaginal microbiome between women with type 1 diabetes who delivered at term (green) and those who delivered prematurely (orange). (**a**) Alpha diversity (InvSimpson) by PTB status and BMI category. Values are model-predicted medians with IQR. Adjusted *p* values were derived from model contrasts comparing PTB status within BMI categories. (**b**) Differentially abundant *L. reuteri*. Values are means with SEM. (**c**) Differentially abundant fungi. *p<0.05, **p<0.01, between women who delivered at term and those who delivered prematurely. (**b**, **c**) Values are means with SEM

PTB was defined as delivery at a gestational age less than 37 weeks. A prior clinical indication was present in 13 participants who had an elective Caesarean section before 37 weeks’ gestation; these were not included in the analyses. For the bacterial analysis 12 samples were excluded leaving a total of 148 women. Of those, 30% (44/148) of women with type 1 diabetes had PTB, compared with 5% (7/150) of those without type 1 diabetes; the low number in the latter group precluded further analysis. In women with type 1 diabetes, alpha diversity analysis (InvSimpson) revealed an interaction between PTB and BMI. Therefore, differences associated with PTB were examined within BMI categories (normal weight: BMI 18.5–24.9 kg/m^2^; overweight: BMI 25–29.9 kg/m^2^; obese: BMI >30 kg/m^2^). Estimated marginal means revealed an inverse relationship in which InvSimpson was significantly greater in normal-weight women with PTB compared to those without PTB (estimated marginal mean difference [original scale] = 1.77, 95% CI 1.21, 2.59; *p*=0.009), whereas in women with obesity it was smaller and not significant (Fig. 3a, Table 3 and ESM Table 5).

No differences were detected for beta diversity associated with PTB (Table 3 and ESM Table 6). For differential abundance, women with type 1 diabetes and PTB had a significant decrease in the abundance of *L. reuteri* (Fig. 3b, Table 4 and ESM Table 7)*.*

For the fungal analysis, eight samples were excluded leaving a total of 62 women. Of those, 27% (17/62) of women with type 1 diabetes had PTB, compared with 2.6% (2/77) of those without type 1 diabetes. In women with type 1 diabetes, no interactions with parity or BMI, or differences in alpha or beta diversity, were detected between those with and without PTB. glmmTMB models revealed a significant enrichment of the genus *Malassezia*, family Malasseziaceae and order Malasseziales in women with type 1 diabetes and PTB (Fig. 3c, Table 4 and ESM Table 7)*.*

#### Pre-eclampsia

**Fig. 4:**
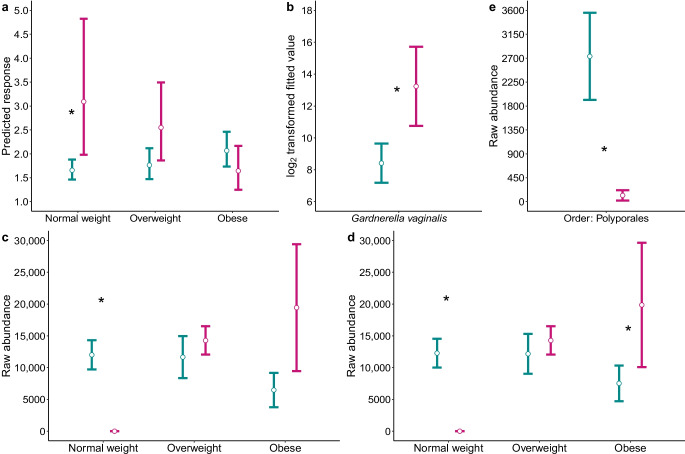
Differences in the vaginal bacterial and fungal microbiome between women with type 1 diabetes who did not develop PE (cyan) and those who did (pink). (**a**) Alpha diversity (InvSimpson) by PE status and BMI category. Values are model-predicted medians with IQR. (**b**) Differentially abundant *G. vaginalis*. (**c**, **d**) Differentially abundant fungi. (**c**) Mean raw abundances and SEM for *Candida albicans* by PE status and BMI category. (**d**) Mean raw abundances and SEM for the *Candida* genus by PE status and BMI category. (**e**) Mean raw abundances and SEM for the Polyporales order by PE status. *p<0.05, between women with type 1 diabetes who did not develop PE and those who did. (**c**–**e**) Values are means with SEM. (**a**, **c**, **d**) Adjusted *p* values were derived from model contrasts comparing PE status within BMI categories

PE was defined by clinical criteria [26]; other causes of hypertension in pregnancy were excluded. PE occurred only in the type 1 diabetes pregnancies, in 18% (29/158) of the type 1 diabetes cohort with available bacterial microbiome data. For alpha diversity, a significant interaction between PE and BMI was detected for InvSimpson; therefore differences due to PE were assessed for each BMI category (ESM Table 5). Women with PE who were of normal weight (estimated marginal mean difference [original scale] = 0.54, 95% CI 0.34, 0.85; adjusted *p*=0.024) or overweight (difference = 0.69, 95% CI 0.48, 1; adjusted *p*=0.07) had higher InvSimpson indices than those without PE (Fig. 4a, Table 3 and ESM Table 5).

**Fig. 5:**
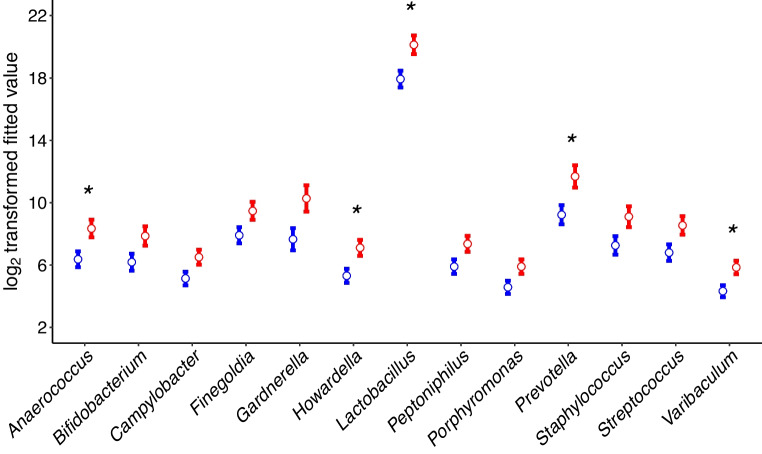
Differentially abundant bacteria between women with and without type 1 diabetes (sensitivity analysis). Differentially abundant bacterial genera in normal-weight women with type 1 diabetes (red) and without (blue), excluding women with type 1 diabetes who had PE. Values are means with SEM. *p<0.05 between women with and without type 1 diabetes

In the beta diversity analysis, no significant differences were detected due to PE (Table 3 and ESM Table 6). However, differential abundance analysis revealed a significant enrichment in the species *G. vaginalis* in type 1 diabetes pregnancies with PE compared to those without PE (Fig. 4b, Table 4 and ESM Table 7). *G. vaginalis* accounted for 100% of the species in the *Gardnerella* genus. Because the abundance of *G. vaginalis* was increased in women with both type 1 diabetes and PE, we performed a sensitivity test to exclude the possibility that the increase of *G. vaginalis* in PE was due to the increased abundance of *Gardnerella* overall in women with type 1 diabetes. Even when women with type 1 diabetes and PE were excluded, *Gardnerella* was still increased (*p*=0.057) in women with type 1 diabetes, supporting the robustness of the association between *Gardnerella* and type 1 diabetes independent of PE status (Fig. 5 and ESM Table 7).

For the fungal analysis, 11/68 (16%) of women with type 1 diabetes developed PE. No differences due to PE were found in the alpha or beta diversity analyses (Table 3, and ESM Tables 4 and 5). glmmTBB models identified an interaction between PE and BMI for the species *C. albicans* and the genus *Candida*. Further pairwise comparisons using the emmeans package examined differences in these two taxa between PE groups within each BMI category. Pairwise comparisons revealed a significant decrease in *C. albicans* and the *Candida* genus among women with PE, but only within the normal-weight group, while the abundance of the *Candida* genus was increased in women with obesity and type 1 diabetes (Fig. 4c and d, Table 4 and ESM Table 7). The order Polyporales was decreased in women with T1D and PE (Fig. 4e).

## Discussion

During pregnancy, the vaginal bacterial microbiome becomes less diverse, with an increase in *Lactobacillus* species, attributed to oestrogen-induced production of glycogen, the main carbohydrate fuel of *Lactobacillus* spp. [2, 3]. We found that the vaginal bacterial microbiome of pregnant women with and without type 1 diabetes was largely dominated by *Lactobacillus* species, particularly *L. crispatus* and *L. iners*. Although we found no differences in the frequency of lactobacilli or specific CSTs between the vaginal microbiomes of women with and without type 1 diabetes, *L. iners* and *L. jensenii* were significantly more abundant in women with type 1 diabetes. This was accompanied by enrichment of anaerobic bacteria such those of the genera *Gardnerella*, *Ureaplasma* and *Prevotella*, which are known to contribute to CST IV, the CST that is associated with the highest pH and lowest protective effect against pathogenic bacteria [1]. Thus, bacterial vaginosis, the most common vaginal infection, mirrors CST IV with a loss of *Lactobacillus* spp. and an increase in anaerobes. Compared with other lactobacilli species in the vaginal microbiome, *L. crispatus* is the most protective against *G. vaginalis* and bacterial vaginosis [27, 28] and PTB [29]. *L. crispatus* was not enriched in women with type 1 diabetes, compared with *L. iners* and* L. jensenii*, which may account for why women with type 1 diabetes have a greater abundance of *Gardnerella* and other anaerobes associated with adverse pregnancy outcomes.

An interesting observation from our study is the lack of significant differences in bacterial alpha diversity between women with and without type 1 diabetes. Although vaginal dysbiosis is often linked to increased diversity and reduced *Lactobacillus* dominance, our results suggest that type 1 diabetes during pregnancy is associated with specific compositional shifts, such as a higher abundance of *L. iners*, *L. jensenii* and anaerobic genera, rather than overall diversity changes. This indicates that the elevated risk of adverse pregnancy outcomes in type 1 diabetes may stem from specific microbiome alterations rather than increased bacterial diversity.

To investigate an association between the vaginal microbiome and adverse pregnancy outcomes, we examined associations with PTB and PE, for which women with type 1 diabetes are at much higher risk [8, 9]. Distinct alterations in the vaginal bacterial microbiome in pregnancy with type 1 diabetes, specifically an increased abundance of the genus *Gardnerella* and other anaerobic bacteria from the *Anaerococcus*, *Dialister*, *Prevotella* and *Peptoniphilus* genera, which are implicated in bacterial vaginosis [30], were associated with these adverse pregnancy outcomes. Bacterial vaginosis has been shown to be associated with an increased risk of PTB [29]. We found a decrease in *L. reuteri* in women with type 1 diabetes and PTB. Thus, the vaginal microbiome associated with PTB may be less protected against colonisation by pathogenic anaerobic bacteria associated with bacterial vaginosis [30].

Normal-weight or overweight women with type 1 diabetes and PE had an increase in alpha diversity compared to those without PE. This is consistent with displacement of *Lactobacillus* spp. as a dominant member of the microbiome by *G. vaginalis*, which is enriched in women with PE, as well as with colonisation by other strict anaerobic species. *G. vaginalis* is a pathogen that is associated with bacterial vaginosis and PTB [29]. A high abundance of *G. vaginalis* in the pregestational vaginal microbiome has been associated with hypertensive disorders in pregnancy [31], and high colony counts of *G. vaginalis* have been found in the urine of women with PE [32]. *G. vaginalis* metabolises glucose and lactate to acetate, succinate and formate [33], leading to a decrease in lactic acid concentration, an increase in pH and a decrease in the growth of lactobacilli and other anaerobic bacteria that induce proinflammatory cytokines [33]. The production of sialidase by *G. vaginalis* may degrade vaginal mucus and, together with the vaginolysin it produces, compromise the vaginal epithelial barrier and further promote inflammation [34]. Increased circulating levels of TNF-α and IL-6 have been reported in women with PE [35] but their origin is uncertain. *G. vaginalis*, within the *Gardnerella* genus, which we found was enriched in women with type 1 diabetes, has been shown to increase the production of IL-6, IL-1β, TNF-α and IL-8 by cultured cervical epithelial cells [34]. Our observations support the view that harbouring *G. vaginalis* at high abundance increases the risk of PE in women with type 1 diabetes. However, if mediated by systemic inflammation secondary to epithelial leakiness, this inflammation may be more likely to emanate from the larger gut microbiome, which we have shown is proinflammatory in pregnant women with type 1 diabetes [36]. Importantly, if the presence of *G. vaginalis* is causally related to pregnancy complications such as PE, its early detection and treatment could decrease risk.

The abundance of *Candida* in the vaginal microbiome varied significantly across groups defined by PE status and BMI. Among normal-weight women with PE, *Candida* was nearly absent, but was enriched in women with obesity and with PE, implying that a deviation from intermediate levels (either depletion or overgrowth) could reflect underlying alterations in the vaginal environment associated with PE. Although speculative, this finding raises the possibility that balanced *Candida* colonisation may be biologically relevant during late pregnancy, playing a role in maintaining vaginal homeostasis.

Type 1 diabetes is strongly linked to HLA class II gene loci on chromosome 6, and HLA genes have been shown to affect the composition of the gut microbiome [37]. However, to our knowledge, no study has examined the relationship between the vaginal microbiome and HLA. Notwithstanding the interaction demonstrated between type 1 diabetes status and HLA, the effect of HLA itself on the vaginal microbiome (beta diversity) was significant in women with and without type 1 diabetes. The effect of type 1 diabetes on the vaginal bacterial microbiome was influenced by HLA type, with significant differences detected for intermediate and low-risk HLA-DR types, but not the highest-risk HLA-DR3,4 type. This finding may reflect an effect of environment acting through the microbiome to promote penetrance of low and intermediate HLA types for type 1 diabetes risk, but not high-risk HLA-DR3,4, as previously suggested [38].

In conclusion, we have identified alterations in the composition of the third trimester vaginal microbiome in women with type 1 diabetes, and their association with two adverse pregnancy outcomes, PTB and PE. However, it is important to place these findings in context. First, the women in this study comprised a randomly selected cohort of white women predominantly of European background who may not be representative of women of other ethnic backgrounds. Second, because the small numbers of PTB and PE cases in the women without type 1 diabetes precluded analysis of these groups, we cannot be certain that our findings apply generally or whether there is an interaction with type 1 diabetes status. Finally, we did not study the vaginal microbiome in early pregnancy when changes may be more distinct and predictive [39]. Nevertheless, ours is the first reported study of adverse pregnancy outcomes in relation to the vaginal microbiome in women with type 1 diabetes. Further research is warranted to explore mechanistic links between the vaginal microbiome and pregnancy outcomes, as a basis for targeted therapy. Meanwhile, interventions to eradicate risk-associated taxa, such as *G. vaginalis* in the case of PE, could improve vaginal health and reduce adverse pregnancy outcomes.

## Supplementary Information

Below is the link to the electronic supplementary material.

ESM (PDF 1.18 MB)

Supplementary file2 (XLSX 78 KB)

## Data Availability

The demultiplexed raw sequencing data supporting the findings of this study are publicly available in the NCBI Sequence Read Archive (SRA; https://www.ncbi.nlm.nih.gov/sra) under project number PRJNA1037097.

## Abbreviations

ASV: Amplicon sequence variants
CST: Community state type
ENDIA: Environmental Determinants of Islet Autoimmunity
ITS1: Internal transcribed spacer
OTU: Operational taxonomic unit
PTB: Pre-term birth
PE: Pre-eclampsia
SH: Species hypotheses
TMM: Trimmed mean of log expression ratios
BMI: Body mass index
HLA: Human leukocyte antigen

## Code availability

All code used in the analyses is available on GitHub at https://github.com/RothSchulze/Adverse-pregnancy-outcomes-in-women-with-T1D-and-vaginal-microbiome, commit 8241215 (archived version used for this analysis). The repository includes Qiime2 command logs for sequence processing, R Markdown scripts and the associated R objects containing taxonomic profiles and metadata used for statistical analyses.
