# Supplementary material for: Adverse pregnancy outcomes in women with type 1 diabetes are associated with multiple alterations in the vaginal microbiome
Source: Diabetologia. 2025 Aug 7;68(11):2552–66. doi: 10.1007/s00125-025-06509-0 (PMC12534357; doi:10.1007/s00125-025-06509-0)
Supplement: Supplementary file 1 — ESM (PDF 1.18 MB) [file 125_2025_6509_MOESM1_ESM.pdf]

Electronic Supplementary Material

Adverse pregnancy outcomes in women with T1D are associated with multiple alterations in the vaginal microbiome

Alexandra J. Roth-Schulze[1], Esther Bandala-Sanchez[1], Katrina M. Ngui[1], Megan A. S. Penno[2], Helena Oakey[2], Enrique Zozaya-Valdes[1], Pat Ashwood[2], Rebecca L. Thomson[2], Dexing Huang[1], Peter G Colman[3], John M Wentworth[1,3], Peter J. Vuillermin[4], Maria E Craig[5], Elizabeth A Davis[6], Anthony Hunyh[7], Georgia Soldatos[8], Jennifer J. Couper[2] and Leonard C. Harrison[1], for the ENDIA Study Group.

[1] Walter and Eliza Hall Institute of Medical Research, Melbourne, VIC 3052, Australia and Department of Medical Biology, University of Melbourne, Melbourne, VIC 3010, Australia.

[2] The University of Adelaide, Robinson Research Institute, Adelaide Medical School, University of Adelaide, Adelaide, SA 5005, Australia.

[3] Department of Diabetes and Endocrinology, Royal Melbourne Hospital, Melbourne, VIC 3050, Australia.

[4] Faculty of School of Medicine, Deakin University and Child Health Research Unit, Barwon Health, Geelong, VIC 3220, Australia.

[5] School of Women's and Children's Health, Faculty of Medicine, University of New South Wales, Sydney, NSW 2052, Australia and Institute of Endocrinology and Diabetes, The Children's Hospital at Westmead, Sydney, NSW 2145, Australia.

[6] Telethon Institute for Child Health Research, Centre for Child Health Research, University of Western Australia, Perth, WA 6009, Australia.

[7] The University of Queensland Diamantina Institute, Faculty of Medicine, University of Queensland, Translational Research Institute, Woolloongabba, QLD 4102, Australia and Queensland Children's Hospital, South Brisbane, QLD 4101, Australia.

[8] Monash Centre for Health Research and Implementation, School of Public Health and Preventive Medicine, Monash University, Melbourne and Diabetes and Vascular Medicine Unit, Monash Health, Melbourne, VIC 3168, Australia.

Corresponding author: Leonard C Harrison, Walter and Eliza Hall Institute of Medical Research, 1G Royal Parade, Parkville, 3052, VIC, Australia; harrison@wehi.edu.au ORCID 0000-0002-2500-8944

Abbreviations

PCoA	Principal Coordinates Analysis
FDR	False discovery rate

Table of Contents

ESM Methods **3**

BLAST features classification *3*

Analysis of vaginal CSTs *3*

Alpha diversity *4*

Beta diversity *5*

Taxa composition analysis *6*

ESM Results **8**

Community State Types (CSTs) *8*

ESM Tables **10**

ESM Table 1. *BLASTn features classifications and representative/most probable species* *10*

ESM Table 2a. *Summary of characteristics of non-T1D and T1D pregnancies in bacterial samples.* *10*

ESM Table 2b. *Summary of characteristics of non-T1D and T1D pregnancies in fungal samples.* *10*

ESM Table 3. *Frequency analysis of Lactobacillus spp* *10*

ESM Table 4. *Frequency analysis of community state types (CSTs).* *11*

ESM Table 5. *Alpha diversity p values. Significant p (<0.05) in bold.* *12*

ESM Table 6a*. Beta diversity comparison for vaginal bacterial and fungal microbiomes.* *13*

ESM Table 6b. *Beta diversity comparison for vaginal bacterial and fungal microbiomes.* *14*

ESM Table 7. *Differential abundance analysis for vaginal bacterial and fungal microbiome.* *14*

ESM Figures **15**

ESM Figure 1. Bacterial microbiome taxonomic composition. *15*

ESM Figure 2. Bacterial community state types (CST) frequency. *15*

ESM Figure 3. Fungal microbiome taxonomic composition. *16*

ESM Figure 4. Alpha diversity. *16*

ESM Figure 5. Beta diversity. *17*

ENDIA Study Group authors 2024 .... **17**

ESM Methods

BLAST features classification

Representative features that could not be classified at the species level were improved by sequence alignment using BLASTn against the NCBI 16S ribosomal RNA database, excluding uncultured/environmental samples. Alignments were filtered to minimum 98% identity, and when possible, the ASVs were assigned to unique species. In the cases where multiple species had the same identity to a sequence, the most likely species was selected based on previously published vaginal data. For example, species *Lactobacillus crispatus* and *L. acidophilus* cannot be distinguished based on the V4 region; however, because *L. crispatus* is the most common species in the vaginal microbiome the sequence was assigned to it. The same applied to *L. gasseri* and *L. johnsonii*, the former being most commonly found in the vaginal microbiome.

Analysis of vaginal CSTs

For the frequency analysis of vaginal CSTs, samples were clustered into six groups based on the dominant OTU classification. Dominance was not defined by a fixed abundance threshold, but rather by the predominance of a particular *Lactobacillus* species relative to other CST-defining taxa (i.e. *Lactobacillus* species, *Bifidobacterium* genus or grouped species abundance from the CST IV) within the microbial community of each individual sample. Nevertheless, consistent with findings from Ravel et al. (2011) and subsequent studies, species dominance corresponds to a relative abundance exceeding 50%. Groups were CST I (*L. crispatus*), CST II (*L. gasseri*), CST III (*L. iners*), CST IV (very few if any *Lactobacilli* and an increase in anaerobic species such as *Gardnerella vaginalis*, *Atopobiun vaginae*, and genera *Prevotella*, *Streptococcus, Dialister, Finegoldia, Mobiluncus, Sneathia, Parvimonas, Fastidiosipila, Peptoniphilus, Anaerococcus, Veillonella* and *Corynebacterium*) and CST V (*L. jensenii*) [1]. In addition, we observed that a few samples (12/310) which were not classified to either of the

aforementioned CSTs, were dominated by bacteria from the genus *Bifidobacterium*. These samples were designated as CST-Bifido. Differences in the frequencies of CSTs were tested by multinomial logistic regression using the R 'mgcv' package add reference "multinom()" function, where the response variable is the CST and the explanatory variable T1D status with covariates parity and body mass index (BMI). Finally, binomial logistic regression was applied using the "glm()" function to test if the distribution (presence/absence) of *Lactobacillus* at a certain percentage is significantly different depending on T1D status. The response variable is presence/absence of *Lactobacillus* (one test for each category >70%, >80% and >90%) and the explanatory variable is T1D status with covariables parity and BMI.

Alpha diversity

Generalized linear mixed models (GLMMs) using the glmmTMB package were applied to evaluate differences in the alpha diversity measures, namely observed richness, Chao1 index, Shannon index and Inverse Simpson index, across groups. For each diversity metric, data distributions were first visually inspected using histograms and formally assessed using the Shapiro-Wilk test to determine the most appropriate statistical family and link function. Based on these assessments, model specifications varied to best accommodate the distributional characteristics of each index: for example, observed richness and Chao1, which often follow count-like or over-dispersed distributions, were modeled using a negative binomial family, while Shannon and Inverse Simpson indices, which are continuous and right-skewed, were modeled using a Gamma distribution with a log link. The model included the exposure variable of interest (i.e., T1D status, gestational age at delivery as a proxy for PTB and PE (hypertension and proteinuria). Random effects were included as necessary to account for repeated measures ("mother ID") and sequencing batches. In addition, the interaction between the exposure variables of interest and the covariates, parity or BMI, was tested and when an interaction was not significant it was removed from the model. When interactions were significant, post hoc

comparisons were conducted using estimated marginal means (EMMs) via the emmeans package [1] v.1.10.5**.** Model selection and goodness-of-fit were evaluated using Akaike's Information Criterion (AIC), residual diagnostics, and checks for overdispersion. All *p* values were adjusted for multiple testing using the Benjamini-Hochberg method. To test for differences in alpha diversity among fungal taxa, the same model selection strategy and functions were applied as in the bacterial analysis. However, the random effect 'sequencing batch' was excluded, as all fungal samples were processed and sequenced in a single batch, and 'mother ID' was also omitted due to the minimal occurrence of repeated measurements.

Beta diversity

Repeated measure aware permutational analysis of variance (RMA-PERMANOVA) of the log transformed and untransformed Bray-Curtis coefficients was performed to determine if differences between groups of interest (i.e. T1D status, PTB and PE) at taxonomic levels, species, genus, family, order and phylum, were significant. In our study, beta diversity was assessed using two approaches: first, with untransformed Bray-Curtis distances, and second, with log-transformed Bray-Curtis distances. The untransformed analysis provides an overview of community composition driven primarily by highly abundant taxa, which may mask subtler differences associated with low-abundance taxa. In contrast, the log-transformed approach down-weights dominant taxa, allowing greater sensitivity to variation among less abundant members of the community. As with the alpha diversity analysis, the statistical models included the exposure variable of interest and adjustments for sequencing batch and mother ID (except in the fungal analysis, where these variables were not applicable). In addition, differences in beta diversity due to human leukocyte antigen (HLA) type were also tested. Interactions between the exposure variable of interest and parity, BMI or HLA were tested and, when not significant, the interaction was removed from the model. Variables for which there was an

interaction were analysed by pairwise comparison using a Benjamini-Hochberg adjustment from the function "pairwise.adonis2" (pairwiseAdonis R package [2]). All *p* values were adjusted for multiple testing using the Benjamini-Hochberg method. Ordination plots were generated using Principal Coordinates Analysis (PCoA) based on log-transformed Bray–Curtis distance matrices to visualize differences in microbial community composition between samples.

Taxa composition analysis

For the bacterial microbiome, a filter was applied before performing the analysis to remove low prevalence taxa (i.e., taxa present in very few samples). Library sizes were normalized using the trimmed mean of log expression ratios with singleton pairing (TMMwsp) method [3] in edgeR, which is expected to perform better for data with a high proportion of zeros. Counts were transformed to log2-counts per million (logCPM), voom precision weights were calculated and limma linear models were fitted while allowing for loss of residual degrees of freedom due to exact zeros using the voomLmFit function [4, 5]. Here, "women IDs" were considered as blocks to calculate the consensus correlation and account for multiple measurements (i.e., same woman, different pregnancy) while estimating contrast statistics using the contrasts.fit function and empirical Bayes moderated t statistics. Where an interaction was detected in the beta diversity analysis, to build our model for the differential abundance analysis we combined the variables that interacted into a single factor and the comparisons of interest were defined as contrasts. Tests included adjustments for parity. In addition, the model design included an adjustment for sample sequencing batch. Adjusted group means were computed before performing the test: for factors, the contr.sum function was used, e.g., contrasts (SequencingBatch) <- contr.sum(levels (SequencingBatch). The mean abundance of each taxon in each group was computed by limma when fitting the model and are contained in the coefficient's component (fit$coef). The standard errors are obtained as follows:

fit$stdev.unscaled(,) * sqrt(fitc$s2.post). Note that after normalization the data are on the log2 scale. The Benjamini-Hochberg method was used to control the false discovery rate (FDR). FDR < 0.1, a common standard for statistical significance in exploratory analyses [6], was considered significant. Because a specific bacterial species was increased in women with compared to women without T1D and the same species was also enriched in women with T1D and preeclampsia compared to women with T1D without preeclampsia, a sensitivity analysis was performed by removing women with both T1D and preeclampsia from the complete data set; the analysis comparing women with and without T1D was then repeated as described above. For HLA, the differential abundance analysis was performed by comparing samples of women with HLA-DR3, 4 (highest risk for T1D) vs. HLA-DRX, X (lowest risk for T1D).

For the fungal microbiome, generalized linear mixed models (GLMMs) using glmmTMB were applied to evaluate differences in the abundance of a subset of 13 taxa across different taxonomic levels (species [2 taxa], genus [2 taxa], family [3 taxa], order [4 taxa], and phylum [2 taxa]). These taxa were preselected from a larger dataset based on a prevalence threshold of >25% across samples, ensuring that only consistently present taxa were included in the models. For each taxon, statistical models with different specifications and structures were implemented to identify the best-fitting model for each case. Model specification refers to the inclusion of different predictor variables and interactions in the model formula (e.g., adjusting for parity and BMI or testing interaction effects), while model structure pertains to the statistical framework used to model the data (e.g., negative binomial vs. zero-inflated models). Given that these variations in model formulation are driven by data-specific properties rather than multiple independent hypothesis tests, applying a multiple testing correction is not necessary in this context. Moreover, multiple testing correction is typically required when performing multiple independent tests on the same hypothesis, increasing the risk of false positives. However, in our study, we did not systematically test the same hypothesis across

multiple taxa with a uniform model. Instead, each taxon was analyzed individually, and the best model was selected based on its distributional characteristics. This approach ensures that the differences in model specification and structure reflect biologically appropriate adjustments rather than multiple independent comparisons requiring correction.

Therefore, given that the subset of taxa was preselected based on a defined prevalence threshold and that the statistical models were tailored individually for each taxon without testing variations of the same hypothesis multiple times, a multiple testing correction was not applied in our differential abundance analysis.

Because of the known effect of parity [7] and BMI [8] on the vaginal microbiome, analysis included the interaction of exposure variables with these covariates; when interactions were not significant, adjustments for the covariates were included in the analysis.

ESM Results

Community State Types (CSTs)

The vaginal bacterial microbiome has been classified into five community state types (CSTs), four of these dominated by *Lactobacillus* species [9]. CSTI is dominated by *Lactobacillus crispatus*, CSTII by *Lactobacillus gasseri*, CSTIII by *Lactobacillus iners* and CSTV by *Lactobacillus jensenii;* CSTIV lacks *Lactobacilli* species and instead comprises a variety of taxa that include *Gardnerella vaginalis*, *Atopobium vaginae*, *Prevotella*, *Streptococcus* and *Dialister*, among others. Additionally, we identified a subset of samples in which bacteria from the *Bifidobacterium* genus were dominant; these were classified as CST-Bifido. In the current study, CSTI was the most frequent CST, present in 43% and 36% of women with and without type 1 diabetes (T1D), respectively, followed by CSTIII, in 25% of women with and without T1D, and CSTIV in 19% and 23% of women with and without T1D (Table E2A and Figure S2).

References

1. Searle SR, Speed FM, Milliken GA (1980) Population Marginal Means in the Linear Model: An Alternative to Least Squares Means. The American Statistician 34(4):216–221. https://doi.org/10.1080/00031305.1980.10483031

2. Martinez Arbizu P (2020) pairwiseAdonis: Pairwise multilevel comparison using adonis. R package version 04 1

3. Robinson MD, Oshlack A (2010) A scaling normalization method for differential expression analysis of RNA-seq data. Genome Biology 11(3):R25. https://doi.org/10.1186/gb-2010-11-3-r25

4. Law CW, Chen Y, Shi W, Smyth GK (2014) voom: precision weights unlock linear model analysis tools for RNA-seq read counts. Genome Biology 15(2):R29. https://doi.org/10.1186/gb-2014-15-2-r29

5. Lun ATL, Smyth GK (2017) No counts, no variance: allowing for loss of degrees of freedom when assessing biological variability from RNA-seq data. Statistical Applications in Genetics and Molecular Biology 16(2):83–93. https://doi.org/10.1515/sagmb-2017-0010

6. Callahan BJ, DiGiulio DB, Goltsman DSA, et al (2017) Replication and refinement of a vaginal microbial signature of preterm birth in two racially distinct cohorts of US women. Proceedings of the National Academy of Sciences 114(37):9966–9971. https://doi.org/10.1073/pnas.1705899114

7. Kervinen K, Holster T, Saqib S, et al (2022) Parity and gestational age are associated with vaginal microbiota composition in term and late term pregnancies. eBioMedicine 81. https://doi.org/10.1016/j.ebiom.2022.104107

8. Allen NG, Edupuganti L, Edwards DJ, et al (2022) The vaginal microbiome in women of reproductive age with healthy weight versus overweight/obesity. Obesity 30(1):142–152. https://doi.org/10.1002/oby.23306

9. Ravel J, Gajer P, Abdo Z, et al (2011) Vaginal microbiome of reproductive-age women. Proceedings of the National Academy of Sciences 108(Supplement_1):4680–4687. https://doi.org/10.1073/pnas.1002611107

ESM Tables

ESM Table 1. BLASTn features classifications and representative/most probable species. See ESM Table 1, only provided separately as Excel file.

ESM Table 2a. Summary of characteristics of non-T1D and T1D pregnancies in bacterial samples. See ESM Table 2a, only provided separately as Excel file.

ESM Table 2b. Summary of characteristics of non-T1D and T1D pregnancies in fungal samples. See ESM Table 2b, only provided separately as Excel file.

ESM Table 3. Frequency analysis of *Lactobacillus* spp.

***Lactobacillus* spp. distribution (presence/absence) between T1D and non-T1D**

Lactobacillus spp.	T1D (160)	Non-T1D (150)
>70%	111 (71%)	98 (68%)
>80%	103 (66%)	93 (64%)
>90%	96 (61%)	80 (55%)

Binomial logistic regression testing the distribution of *Lactobacillus* at different relative abundances - unadjusted *p* values.

Lactobacillus spp.	T1D
>70%	0.56
>80%	0.79
>90%	0.29

***Lactobacillus* spp. distribution between T1D and non-T1D, and parity - Number of samples (percentage of samples)**

	T1D			Non-T1D		
Lactobacillus spp.	Nullipara (85)	Multipara (75)	All (160)	Nullipara (68)	Multipara (82)	All (150)
>70%	63 (76%)	48 (65%)	111 (71%)	50 (78%)	48 (59%)	98 (68%)
>80%	61 (73%)	42 (57%)	103 (66%)	48 (75%)	45 (56%)	93 (64%)
>90%	57 (69%)	39 (53%)	96 (61%)	42 (66%)	38 (47%)	80 (55%)

***Lactobacillus* spp. distribution between T1D and non-T1D and BMI - Number of samples (percentage of samples)**

Lactobacillus spp.	Normal (67)	Obese (45)	Overweight (42)	Underweight (3)	All (157)
>70% - T1D	47 (70%)	30 (67%)	32 (76%)	2 (67%)	111 (71%)
>80% - T1D	45 (67%)	27 (60%)	29 (69%)	2 (67%)	103 (66%)
>90% - T1D	40 (60%)	25 (56%)	29 (69%)	2 (67%)	96 (61%)
	**Normal (81)**	**Obese (30)**	**Overweight (32)**	**Underweight (2)**	**All (145)**
>70% - Non-T1D	57 (70%)	18 (60%)	21 (66%)	2 (100%)	98 (68%)
>80% - Non-T1D	54 (67%)	17 (57%)	20 (62%)	2 (100%)	93 (64%)
>90% - Non-T1D	48 (59%)	13 (43%)	17 (53%)	2 (100%)	80 (55%)

Binomial logistic regression testing the distribution of *Lactobacillus* among T1D status at different relative abundances (interaction with parity and BMI) - adjusted *p* values.

Lactobacillus spp.	Parity	BMI	T1D	Parity:T1D	BMI:T1D
>70%	**0.01**	0.86	0.70	0.51	0.64
>80%	**0.002**	0.81	0.97	0.81	0.69
>90%	**0.007**	0.52	0.39	0.94	0.54

T1D: Type 1 diabetes status
BMI: Body mass index
BMI:T1D; Interaction between BMI and T1D status
Parity:T1D; Interaction between parity and T1D status

Table additionally provided in ESM Excel file

ESM Table 4. Frequency analysis of community state types (CSTs).

CST distribution between T1D and non-T1D (each CST separately) - Number of samples (percentage of samples)

	T1D	Non-T1D	All
CST-Bifido	7 (4%)	5 (3%)	12 (4%)
CSTI	69 (43%)	54 (36%)	123 (40%)
CSTII	6 (4%)	13 (9%)	19 (6%)
CSTIII	39 (24%)	37 (25%)	76 (25%)
CSTIV	33 (21%)	34 (23%)	67 (22%)
CSTV	6 (4%)	7 (5%)	13 (4%)
Total	160	150	310

Multinomial logistic regression testing differences in frequencies of CST among T1D status (all CSTs together) - Unadjusted *p* value

	T1D
CSTI	0.88
CSTII	0.15
CSTIII	0.65
CSTIV	0.56
CSTV	0.54

CST distribution between T1D and non-T1D women separated by parity - Number of samples (percentage of samples)

	T1D			Non-T1D		
Parity	Nulli	Multi	All	Nulli	Multi	All
CST-Bifido	3 (4%)	4 (5%)	7 (4%)	1 (1%)	4 (5%)	5 (3%)
CSTI	48 (56%)	21 (28%)	69 (43%)	42 (62%)	12 (15%)	54 (35%)
CSTII	1 (1%)	5 (7%)	6 (4%)	1 (1%)	12 (15%)	13 (9%)
CSTIII	16 (19%)	23 (31%)	39 (24%)	11 (16%)	26 (32%)	37 (25%)
CSTIV	15 (18%)	18 (24%)	33 (21%)	10 (15%)	24 (29%)	34 (23%)
CSTV	2 (2%)	4 (5%)	6 (4%)	3 (4%)	4 (5%)	7 (5%)
Total	85	75	160	68	82	150

Distribution of CSTs between women with and without T1D separated by BMI categories - Number of samples (percentage of samples)

BMI	Normal (67)	Obese (45)	Overweight (42)	Underweight (3)	All (157)
CST-Bifido - T1D	4 (6%)	2 (4%)	1 (2%)	0 (0%)	7 (4%)
CSTI - T1D	33 (49%)	16 (36%)	18 (43%)	0 (0%)	67 (43%)
CSTII - T1D	3 (4%)	2 (4%)	1 (2%)	2 (0%)	6 (4%)
CSTIII - T1D	9 (13%)	14 (31%)	14 (33%)	2 (67%)	39 (25%)
CSTIV - T1D	14 (21%)	10 (22%)	7 (17%)	1 (33%)	32 (20%)
CSTV -T1D	4 (6%)	1 (2%)	1 (2%)	0 (0%)	6 (4%)
	Normal (81)	Obese (30)	Overweight (32)	Underweight (2)	All (145)
CST-Bifido - Non- T1D	3 (4%)	0 (0%)	2 (6%)	0 (0%)	5 (3%)
CSTI - Non-T1D	31 (38%)	9 (30%)	11 (34%)	0 (0%)	51 (35%)
CSTII - Non-T1D	7 (9%)	4 (13%)	2 (6%)	0 (0%)	13 (9%)
CSTIII - Non-T1D	23 (28%)	5 (17%)	8 (25%)	1 (50%)	37 (26%)
CSTIV - Non-T1D	14 (17%)	11 (37%)	7 (22%)	0 (0%)	32 (22%)
CSTV - Non-T1D	3 (4%)	1 (3%)	2 (6%)	1 (50%)	7 (5%)

Multinommial logistic regression test of differences in frequencies of CST among T1D status an interaction with BMI and parity - adjusted *p* values

	Parity	BMI	T1D	Parity:T1D	BMI:T1D
CSTI	**0.02**	0.35	0.88	0.22	0.63
CSTII	0.54	0.60	0.81	0.86	0.95
CSTIII	0.62	0.79	0.52	0.67	0.70
CSTIV	0.49	0.21	0.72	0.65	0.54
CSTV	0.38	0.71	0.82	0.37	0.89

Table additionally provided in ESM Excel file

ESM Table 5. Alpha diversity *p* values. Significant *p* (<0.05) in bold.

	Exposure variable	Adjustments		Significant interaction (P-	Comparison within levels of variable with	*p*	Adjusted *p*	Estimate^	95% Confidence interval^	
		Fixed	Random						2.50%	97.50%
**Bacterial Richness**	T1D status	Parity, BMI	SeqBatch, motherID	..	..	0.85	0.92	1	0.85	1.15
	Pre-term birth*	Parity, BMI	SeqBatch, motherID	..	..	0.94	0.94	1	0.76	1.28
	Preeclampsia*	Parity, BMI	SeqBatch, motherID	..	..	0.65	0.65	1.06	0.81	1.41
**Bacterial Chao1**	T1D status	Parity, BMI	SeqBatch, motherID	..	..	0.92	0.92	1	0.85	1.16
	Pre-term birth*	Parity, BMI	SeqBatch, motherID	..	..	0.9	0.94	0.98	0.75	1.28
	Preeclampsia*	Parity, BMI	SeqBatch, motherID	..	..	0.65	0.65	1.07	0.81	1.43
**Bacterial InvSimpson**	T1D status	Parity, BMI	SeqBatch, motherID	..	..	0.71	0.92	1.02	0.91	1.16
	Pre-term birth*	Parity, BMI	SeqBatch, motherID	Pre-term birth:BMI (**0.002**)	Normal weight	**0.003**	**0.009**	1.77	1.21	2.59
					Overweight	0.74	0.74	1.06	0.76	1.46
					Obese	0.07	0.11	0.75	0.55	1.02
	Preeclampsia*	Parity, BMI	SeqBatch, motherID	Preeclampsia:BMI (**0.005**)	Normal weight	**0.008**	**0.024**	0.54	0.34	0.85
					Overweight	**0.047**	0.071	0.69	0.48	1
					Obese	0.17	0.17	1.26	0.91	1.74
**Bacterial shannon**	T1D status	Parity, BMI	SeqBatch, motherID	..	..	0.87	0.92	1.02	0.81	1.28
	Pre-term birth*	Parity, BMI	SeqBatch, motherID	..	..	0.52	0.94	0.88	0.59	1.29
	Preeclampsia*	Parity, BMI	SeqBatch, motherID	..	..	0.24	0.65	1.3	0.85	2.04
**Fungal Richness**	T1D status	Parity, BMI	..	..	..	0.44	0.79	1.1	0.86	1.41
	Pre-term birth*	Parity, BMI	..	..	..	0.21	0.37	0.74	0.46	1.18
	Preeclampsia*	Parity, BMI	..	..	..	0.88	1	0.96	0.58	1.64
**Fungal Chao1**	T1D status	Parity, BMI	..	..	..	0.45	0.79	1.1	0.85	1.42
	Pre-term birth*	Parity, BMI	..	..	..	0.28	0.37	0.76	0.46	1.23
	Preeclampsia*	Parity, BMI	..	..	..	0.79	1	0.93	0.56	1.65
**Fungal InvSimpson**	T1D status	Parity, BMI	..	..	..	0.64	0.79	1.05	0.85	1.31
	Pre-term birth*	Parity, BMI	..	..	..	0.22	0.37	0.76	0.48	1.17
	Preeclampsia*	Parity, BMI	..	..	..	0.41	1	0.82	0.53	1.35
**Fungal Shannon**	T1D status	Parity, BMI	..	..	..	0.79	0.79	1.06	0.7	1.58
	Pre-term birth*	Parity, BMI	..	..	..	0.56	0.56	0.79	0.35	1.76
	Preeclampsia*	Parity, BMI	..	..	..	0.81	1	0.89	0.35	2.19

T1D status: Type 1 diabetes status; levels: T1D, Non-T1D
Pre-term birth levels: Pre-term, Term
Preeclampsia levels: Preeclampsia, Non-Preeclampsia
Parity levels: Nulliparous, multiparous
BMI: Body mass index; levels: Normal weight, overweight, obese
Note: Models for testing differences in bacterial diversity were fit using lme4 -glmer.nb, while MASS - glm.nb was used for testing fungal diversity.
Note: p-values for normal, overweight and obese were calculated via contrasts
* Analysed only in samples from women with T1D
^ Original scale (i.e. Observed taxa for richness or Inverted Simpson index

Table additionally provided in ESM Excel file

ESM Table 6a. Beta diversity comparison for vaginal bacterial and fungal microbiomes. Log transformed data. Significant *p* (<0.05) in bold.

	Exposure variable	Adjustments		Taxonomic level	Significant interaction (P-value)	Comparison within levels of variables with interaction	*p*	Adjusted *p*	R2	R2 95% Confidence interval
		Fixed	Random							
Bacterial Beta diversity	T1D status	Parity, BMI	SeqBatch, motherID	Species	..	..	0.39	0.39	0.32%	-0.92%; 1.56%
				Genus	..	..	0.24	0.39	0.46%	-1.03%; 1.94%
				Family	..	..	0.22	0.39	0.51%	-1.05%; 2.07%
				Order	..	..	0.33	0.39	0.41%	-0.99%; 1.81%
				Phylum	..	..	0.27	0.39	0.44%	-1.01%; 1.89%
	Pre-term birth*	Parity, BMI	SeqBatch, motherID	Species	..	..	0.51	0.54	0.16%	-1.0.7%; 1.40%
				Genus	..	..	0.52	0.54	0.47%	-1.63%;2.56%
				Family	..	..	0.54	0.54	0.36%	-1.48%; 2.19%
				Order	..	..	0.52	0.54	0.37%	-1.5%; 2.2%
				Phylum	..	..	0.52	0.54	0.48%	-1.65%; 2.62%
	Preeclampsia*	Parity, BMI	SeqBatch, motherID	Species	..	..	0.27	0.3	0.52%	-1.62%; 2.66%
				Genus	..	..	0.28	0.3	1.14%	-2.01%; 4.29%
				Family	..	..	0.29	0.3	1.41%	-2.09%; 4.92%
				Order	..	..	0.3	0.3	1.51%	-2.1%; 5.13%
				Phylum	..	..	0.27	0.3	0.84%	-1.87%; 3.55%
	HLA type	T1D, Parity, BM	SeqBatch, motherID	Species	HLA:T1D (0.026)	T1D (HLA-DR34 vs HLA-DRXX)	0.24	0.4	1.90%	-4%; 7.9%
						Non-T1D (HLA-DR34 vs HLA-DRXX)	0.41	0.51	1.52%	-4%; 7.1%
						HLA-DRXX (T1D vs Non-T1D)	0.056	0.14	3.20%	-4.5%; 11%
						HLA-DR Group3o4 (T1D vs Non-T1D)	**0.046**	0.14	1.30%	-2%; 4.7%
						HLA-DR34 (T1D vs Non-T1D)	0.86	0.86	0.78%	-3.2%; 4.7%
				Genus	HLA:T1D (0.001)	T1D (HLA-DR34 vs HLA-DRXX)	**0.045**	0.058	3.70%	-4.4%; 12%
						Non-T1D (HLA-DR34 vs HLA-DRXX)	**0.046**	0.058	4.08%	-4.7%; 13%
						HLA-DRXX (T1D vs Non-T1D)	**0.003**	**0.015**	10.34%	-2.5%; 23%
						HLA-DR Group3o4 (T1D vs Non-T1D)	**0.007**	**0.018**	2.84%	-2.1%; 7.8%
						HLA-DR34 (T1D vs Non-T1D)	0.67	0.67	1.10%	-3.6%; 5.8%
				Family	HLA:T1D (0.001)	T1D (HLA-DR34 vs HLA-DRXX)	**0.046**	0.064	4.00%	-4.4%; 12%
						Non-T1D (HLA-DR34 vs HLA-DRXX)	0.051	0.064	4.00%	-4.8%; 13%
						HLA-DRXX (T1D vs Non-T1D)	**0.002**	**0.01**	12.00%	-1.5%; 26%
						HLA-DR Group3o4 (T1D vs Non-T1D)	**0.005**	**0.013**	3.26%	-2%; 8.5%
						HLA-DR34 (T1D vs Non-T1D)	0.63	0.63	1.05%	-3.5%; 5.6%
				Order	HLA:T1D (0.001)	T1D (HLA-DR34 vs HLA-DRXX)	0.054	0.082	4.24%	-4.4%; 13%
						Non-T1D (HLA-DR34 vs HLA-DRXX)	0.066	0.082	4.37%	-4.7%; 13%
						HLA-DRXX (T1D vs Non-T1D)	**0.001**	**0.005**	13.00%	-1%; 27%
						HLA-DR Group3o4 (T1D vs Non-T1D)	**0.008**	**0.02**	2.91%	-2.1%; 7.9%
						HLA-DR34 (T1D vs Non-T1D)	0.62	0.62	1.00%	-3.4%; 5.4%
				Phylum	HLA:T1D(0.001)	T1D (HLA-DR34 vs HLA-DRXX)	0.082	0.14	4.08%	-4.4%; 12.6%
						Non-T1D (HLA-DR34 vs HLA-DRXX)	0.13	0.16	3.47%	-4.7%; 11.7%
						HLA-DRXX (T1D vs Non-T1D)	**0.007**	**0.018**	13.00%	-1%; 27.5%
						HLA-DR Group3o4 (T1D vs Non-T1D)	**0.003**	**0.015**	4.29%	-1.7%; 10%
						HLA-DR34 (T1D vs Non-T1D)	0.85	0.85	0.20%	-1.8%; 2.2%
Fungal Beta diversity	T1D status	Parity, BMI	SeqBatch, motherID	Species	..	..	**0.044**	0.073	1.36%	-2.29%; 5%
				Genus	..	..	**0.034**	0.073	1.44%	-2.31%; 5.19%
				Family	..	..	**0.013**	0.065	1.63%	-2.36%; 5.61%
				Order	..	..	0.53	0.58	0.59%	-1.83%; 3%
				Phylum	..	..	0.58	0.58	0.18%	-1.17%; 1.53%
	Pre-term birth*	Parity, BMI	SeqBatch, motherID	Species	..	..	0.48	0.75	1.50%	-4.1%; 7.1%
				Genus	..	..	0.57	0.75	1.44%	-4.05%; 6.93%
				Family	..	..	0.6	0.75	1.40%	-4.05%; 6.93%
				Order	..	..	0.38	0.75	1.82%	-4.33%; 7.96%
				Phylum	..	..	0.82	0.82	0.01%	-0.32%; 0.33%
	Preeclampsia*	Parity, BMI	SeqBatch, motherID	Species	..	..	0.63	0.71	1.21%	-3.5%; 6%
				Genus	..	..	0.62	0.71	1.20%	-3.5%; 5.9%
				Family	..	..	0.66	0.71	1.17%	-3.5%; 5.8%
				Order	..	..	0.27	0.71	1.94%	-4%; 7.9%
				Phylum	..	..	0.71	0.71	0.21%	-1.8%; 2.2%
	HLA Type	T1D, Parity, BM	SeqBatch, motherID	Species	..	..	0.4	0.75	1.47%	-2.6%; 5.3%
				Genus	..	..	0.42	0.75	1.48%	-2.4%; 5.3%
				Family	..	..	0.45	0.75	1.44%	-2.4%; 5.2%
				Order	..	..	0.7	0.88	1.15%	-2.3%; 4.6%
				Phylum	..	..	0.98	0.98	0.00%	-0%; 0%

T1D status: Type 1 diabetes status; levels: T1D, Non-T1D
Pre-term birth levels: Pre-term, Term
Preeclampsia levels: Preeclampsia, Non-Preeclampsia
Parity levels: Nulliparous, multiparous
BMI: Body mass index; levels: Normal weight, overweight, obese
HLA: Human leucocyte antigen; levels: HLA-DR34, HLA-DR Group3o4 and HLA-DRXX
HLA-DR Group3o4: any combination of DR 3 and 4, but not 3,4, i.e., HLA-DR 3, X, -DR 4, X, -DR 3, 3, -DR 4, 4
Note: Models for testing differences beta diversity were fit using Vegan - PERMANOVA
* Analysed only in samples from women with T1D

Table additionally provided in ESM Excel file

ESM Table 6b. Beta diversity comparison for vaginal bacterial and fungal microbiomes. Untransformed data. Significant *p* (<0.05) in bold.

	Exposure variable	Adjustments: Fixed	Adjustments: Random	Taxonomic level	Significant interaction (P-value)	Comparison within levels of variables with interaction	*p*	Adjusted *p*	R2	R2 95% Confidence interval
Bacterial Beta diversity	T1D status	Parity, BMI	SeqBatch, motherID	Species	T1D status : BMI (0.001)	Normal weight (T1D vs. Non-T1D)	0.13	0.29	1.12%	-2.1%; 4.4%
						Overweight (T1D vs. Non-T1D)	0.33	0.33	1.25%	-3.4%; 5.9%
						Obese (T1D vs. Non-T1D)	0.19	0.29	1.81%	-3.7%; 7.4%
				Genus	..	..	0.36	0.36	0.36%	-0.95%; 1.64%
				Family	..	..	0.3	0.36	0.36%	-0.95%; 1.67%
				Order	..	..	0.32	0.36	0.37%	-0.97%; 1.7%
				Phylum	..	..	0.22	0.36	0.46%	-1.03%; 1.96%
	Pre-term birth*	Parity, BMI	SeqBatch, motherID	Species	..	..	0.77	0.81	0.54%	-1.7%; 2.80%
				Genus	..	..	0.81	0.81	0.57%	-1.78%;2.92%
				Family	..	..	0.57	0.81	0.41%	-1.58%; 2.4%
				Order	..	..	0.53	0.81	0.45%	-1.64%; 2.55%
				Phylum	..	..	0.52	0.81	0.39%	-1.56%; 2.35%
	Preeclampsia*	Parity, BMI	SeqBatch, motherID	Species	..	..	0.32	0.52	0.81%	-1.92%; 3.54%
				Genus	Preeclampsia : BMI (0.023)	Normal weight (PE vs. Non-PE)	0.17	0.26	2.12%	-4.16%; 8.4%
						Overweight (PE vs. Non-PE)	0.07	0.21	6.56%	-6.32%; 19.5%
						Obese (PE vs. Non-PE)	0.61	0.61	1.53%	-4.8%; 7.87%
				Family	Preeclampsia : BMI (0.026)	Normal weight (PE vs. Non-PE)	0.34	0.51	1.57%	-3.86%; 4.37%
						Overweight (PE vs. Non-PE)	0.072	0.22	6.07%	-6.38%; 18.5%
						Obese (PE vs. Non-PE)	0.51	0.51	1.63%	-4.9%; 8.15%
				Order	Preeclampsia : BMI (0.029)	Normal weight (PE vs. Non-PE)	0.31	0.47	1.74%	-2.13%; 7.44%
						Overweight (PE vs. Non-PE)	0.08	0.24	6.20%	-6.37%; 18.77%
						Obese (PE vs. Non-PE)	0.57	0.57	1.57%	-4.84%; 7.99%
				Phylum	..	..	0.52	0.52	1.50%	-2.18%; 5.18%
	HLA type	Г1D, Parity, BMI	SeqBatch, motherID	Species	HLA:T1D (0.043)	T1D (HLA-DR34 vs HLA-DRXX)	0.1	0.23	2.51%	-4.3%; 9.3%
						Non-T1D (HLA-DR34 vs HLA-DRXX)	0.75	91	0.74%	-3.2%; 4.6%
						HLA-DRXX (T1D vs Non-T1D)	0.06	0.23	3.49%	-4.5%; 11.5%
						HLA-DRGroup3o4 (T1D vs Non-T1D)	0.14	0.23	0.99%	-2%; 3.9%
						HLA-DR34 (T1D vs Non-T1D)	0.91	0.91	0.50%	-2.7%; 3.7%
				Genus	HLA:T1D (0.001)	T1D (HLA-DR34 vs HLA-DRXX)	**0.006**	**0.015**	7.20%	-3.7%; 18.2%
						Non-T1D (HLA-DR34 vs HLA-DRXX)	0.27	0.34	2.20%	-4.4%; 8.7%
						HLA-DRXX (T1D vs Non-T1D)	**0.001**	**0.005**	13.22%	-0.8%; 27%
						HLA-DRGroup3o4 (T1D vs Non-T1D)	**0.009**	**0.015**	2.34%	-2.1%; 6.8%
						HLA-DR34 (T1D vs Non-T1D)	0.61	0.61	1.13%	-3.6%; 5.7%
				Family	HLA:T1D (0.001)	T1D (HLA-DR34 vs HLA-DRXX)	**0.014**	**0.025**	7.39%	-3.7%; 18.4%
						Non-T1D (HLA-DR34 vs HLA-DRXX)	0.222	0.28	2.25%	-4.4%; 8.9%
						HLA-DRXX (T1D vs Non-T1D)	**0.001**	**0.005**	14.88%	-0.29%; 29.5%
						HLA-DRGroup3o4 (T1D vs Non-T1D)	**0.015**	**0.025**	2.66%	-2.1%; 7.4%
						HLA-DR34 (T1D vs Non-T1D)	0.61	0.61	1.13%	-3.6%; 5.9%
				Order	HLA:T1D (0.001)	T1D (HLA-DR34 vs HLA-DRXX)	**0.01**	**0.025**	7.32%	-3.7%; 18.3%
						Non-T1D (HLA-DR34 vs HLA-DRXX)	0.24	0.3	2.28%	-4.4%; 9%
						HLA-DRXX (T1D vs Non-T1D)	**0.001**	**0.005**	15.05%	-0.41%; 29.7%
						HLA-DRGroup3o4 (T1D vs Non-T1D)	**0.015**	**0.025**	2.77%	-2.1%; 7.6%
						HLA-DR34 (T1D vs Non-T1D)	0.56	0.56	1.06%	-3.5%; 5.7%
				Phylum	HLA:T1D(0.001)	T1D (HLA-DR34 vs HLA-DRXX)	**0.01**	**0.017**	7.33%	-3.7%; 18.3%
						Non-T1D (HLA-DR34 vs HLA-DRXX)	0.51	0.64	1.07%	-3.6%; 5.7%
						HLA-DRXX (T1D vs Non-T1D)	**0.002**	**0.01**	14.78%	0.22%; 29.3%
						HLA-DRGroup3o4 (T1D vs Non-T1D)	**0.005**	**0.013**	3.86%	-1.8%; 9.5%
						HLA-DR34 (T1D vs Non-T1D)	0.68	0.68	0.52%	-2.7%; 3.8%
Fungal Beta diversity	T1D status	Parity, BMI	SeqBatch, motherID	Species	..	..	0.096	0.16	1.15%	-2.2%; 4.5%
				Genus	..	..	0.094	0.16	1.10%	-2.19%; 4.39%
				Family	..	..	0.055	0.16	1.24%	-2.23%; 4.74%
				Order	..	..	0.72	0.72	0.46%	-1.69%; 2.61%
				Phylum	..	..	0.66	0.72	0.19%	-1.18%; 1.55%
	Pre-term birth*	Parity, BMI	SeqBatch, motherID	Species	..	..	0.53	0.66	1.42%	-4.1%; 6.9%
				Genus	..	..	0.42	0.66	1.72%	-4.3%; 7.74%
				Family	..	..	0.39	0.66	1.66%	-4.26%; 7.59%
				Order	..	..	0.28	0.66	2.14%	-4.55%; 8.84%
				Phylum	..	..	0.66	0.66	0.40%	-2.55%; 3.36%
	Preeclampsia*	Parity, BMI	SeqBatch, motherID	Species	..	..	0.79	0.79	1.07%	-3.5%; 5.64%
				Genus	..	..	0.79	0.79	1.05%	-3.5%; 5.6%
				Family	..	..	0.77	0.79	1.00%	-3.5%; 5.5%
				Order	..	..	0.34	0.79	1.70%	-4.05%; 7.4%
				Phylum	..	..	0.64	0.79	0.43%	-2.5%; 3.3%
	HLA Type	Г1D, Parity, BMI	SeqBatch, motherID	Species	..	..	0.34	0.51	1.56%	-2.4%; 5.5%
				Genus	..	..	0.3	0.51	1.56%	-2.4%; 5.5%
				Family	..	..	0.36	0.51	1.51%	-2.4%; 5.4%
				Order	..	..	0.41	0.51	1.47%	-2.3%; 4.6%
				Phylum	..	..	0.73	0.73	0.52%	-0.2%; 5.3%

T1D status: Type 1 diabetes status; levels: T1D, Non-T1D
Pre-term birth levels: Pre-term, Term
Preeclampsia levels: Preeclampsia, Non-Preeclampsia
Parity levels: Nulliparous, multiparous
BMI: Body mass index; levels: Normal weight, overweight, obese
HLA: Human leucocyte antigen; levels: HLA-DR34, HLA-DRGroup3o4 and HLA-DRXX
HLA-DRGroup3o4: any combination of DR 3 and 4, but not 3,4, i.e., HLA-DR 3, X, -DR 4, X, -DR 3, 3, -DR 4, 4
Note: Models for testing differences beta diversity were fit using Vegan - PERMANOVA
* Analysed only in samples from women with T1D

Table additionally provided in ESM Excel file

ESM Table 7. Differential abundance analysis for vaginal bacterial and fungal microbiome. See ESM Table 7, only provided separately as Excel file.

ESM Figures

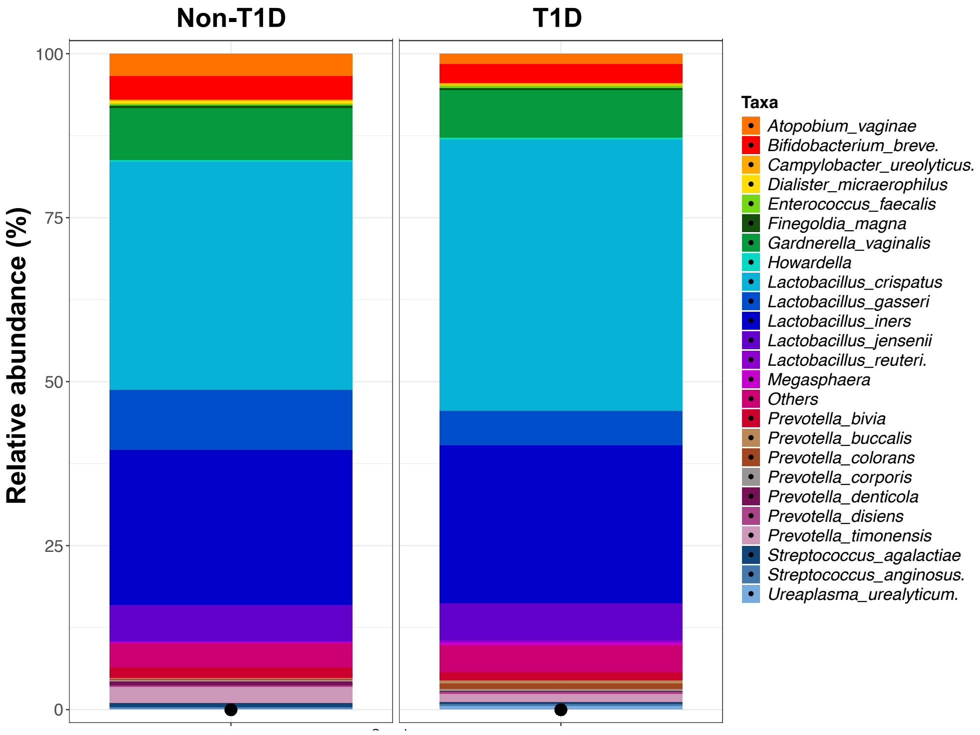

ESM Figure 1. Bacterial microbiome taxonomic composition.
Taxonomic composition of the 25 most abundant bacterial species in vaginal swab samples collected in the third trimester of 310 pregnancies (160 with T1D).

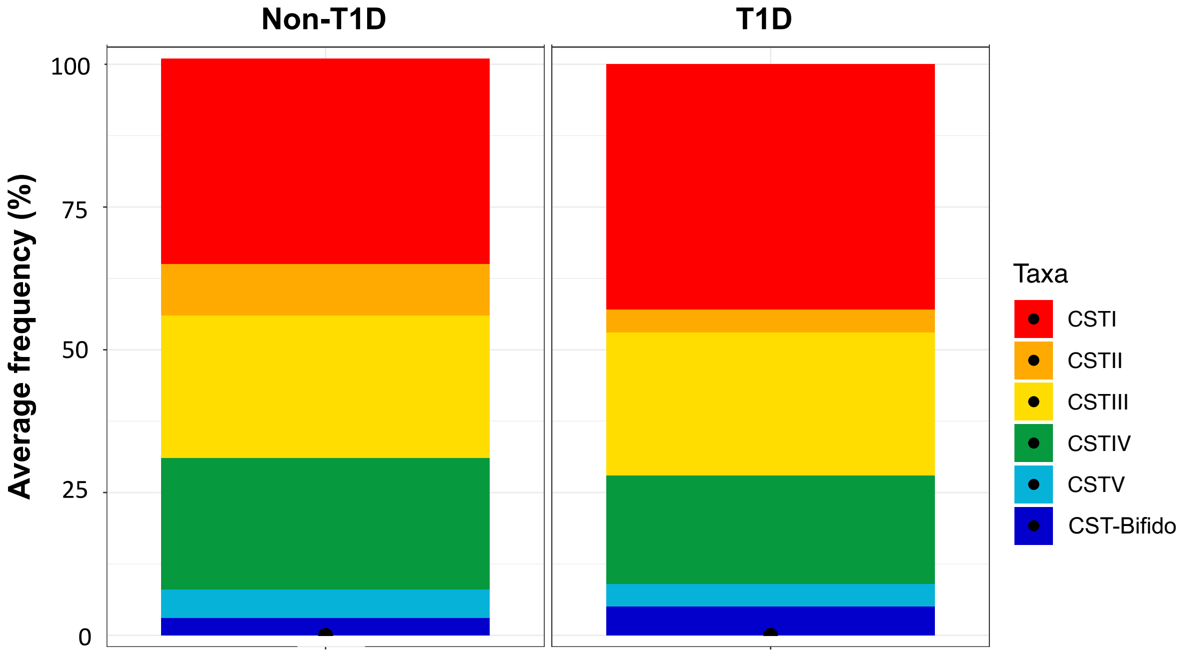

ESM Figure 2. Bacterial community state types (CST) frequency.
Bacterial community state types (CSTs) frequency in vaginal samples from women without and with T1D.

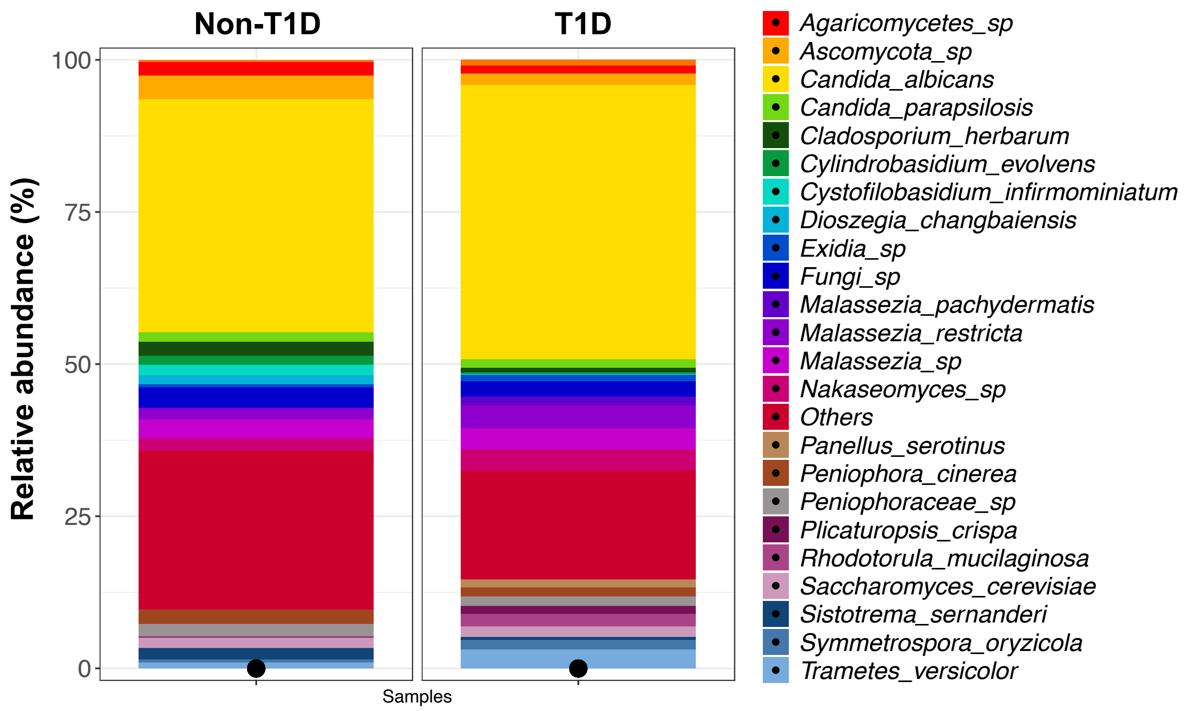

ESM Figure 3. Fungal microbiome taxonomic composition.
The 25 most abundant fungal species in vaginal samples collected in trimester 3 of 147 pregnancies (70 with T1D). T1D: Type 1 diabetes.

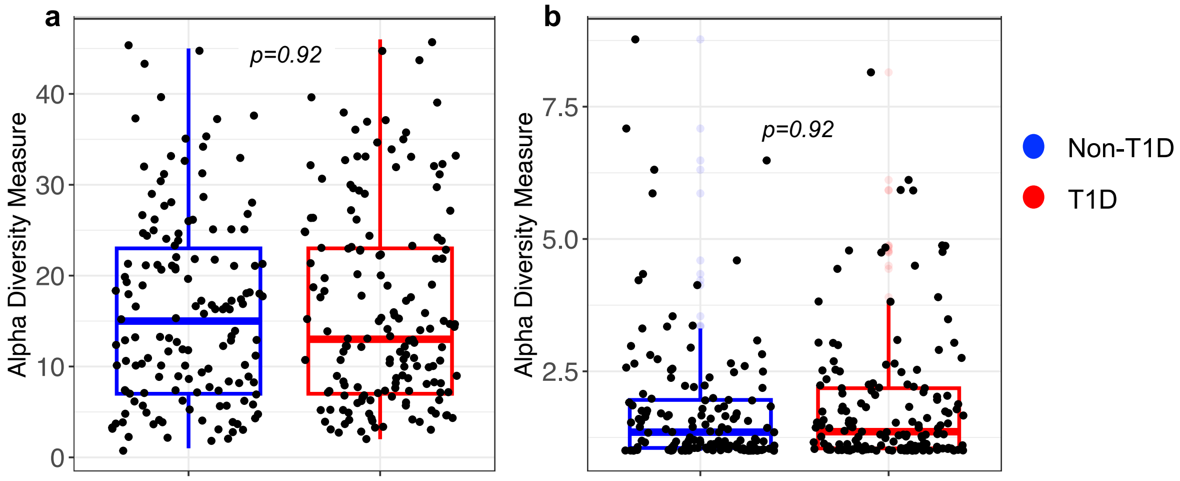

ESM Figure 4. Alpha diversity.
Alpha diversity of the vaginal bacterial microbiome divided by T1D status. Figure a) shows observed richness while b) shows InvSimpson index values. Shown are medians with interquartile range. *p*= Adjusted *p* value.

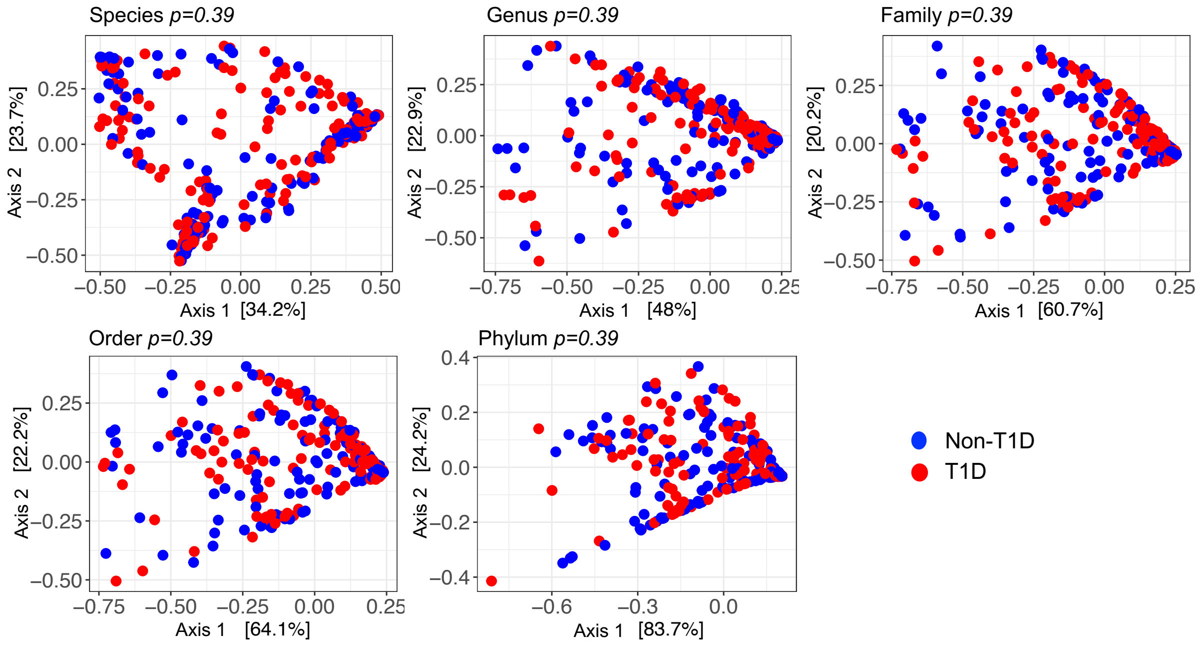

ESM Figure 5. Beta diversity.
Beta diversity of the vaginal bacterial microbiome by T1D status. PCoA ordination plots based on Bray-Curtis distances between samples at the species, genus, family, order and phylum taxonomic levels. *p*= Adjusted *p* value.

ENDIA Study Group authors 2024

Investigators: Simon C Barry, Maria E Craig, Peter G Colman, Jennifer J Couper, Elizabeth A Davis, Emma Hamilton-Williams, Leonard C Harrison, Aveni Haynes, Tony Huynh, Ki Wook Kim, Grant Morahan, Helena Oakey, Megan A S Penno, William D Rawlinson, Richard O Sinnott, Georgia Soldatos, Rebecca L Thomson, Jason Tye-Din, Peter J Vuillermin, John M Wentworth. *Associate Investigators*: Fergus Cameron, Andrew Day, Prudence Lopez. *Project, Data and Biospecimen Managers*: Amanda J Anderson, Pat Ashwood, James D Brown, William Hu, Dao Huynh, Kelly J McGorm. *Clinical scientists*: Kelly Watson. *Coordinators*: Sarah Beresford, Debra Bezuidenhout, Susan Brandrick, Carlie Butterworth, Jacki Catteau, Helen Griffiths, Alison Gwiazdzinski, Candice Hall, Amanda Hulley, Lee Henneken, Renee Kludas, Ying Mateevici, Benjamin Ramoso, Alison Roberts, Alexandra Tully, Rosemary

Wood. *Research Officers*: Sabrina Binkowski, Minh Bui, Abbey Gilbert, Dexing Huang, Ana Karceva, Brydie-Rose Mellor, Gaetano Naselli, Katrina Ngui, Trung Nguyen, Bina Patel, Vanessa Prajitno, Natalie Stone, Thao Tran, Sapphire Vaega, Emily Ward, Yan Xu, Cynthia Yau. *Dietitians*: Rachel Battersby. *Post-doctoral Fellows*: Bek Brittain, Charles Foster, Christopher Hope, Preston Leung, Kylie-Ann Mallitt, Alexandra Roth-Schulze, Tim Sadlon, Bree Tillett, Gregory Walker, Ying Ying Wong, Enrique Zozaya-Valdes. *Administrator*: Leanne Cavenett.

The ENDIA Study Group would like to thank the following institutions and individuals for their contribution to ENDIA recruitment and follow-up:

Lead Clinical Recruitment/Follow-up Sites: The Women's and Children's Hospital, SA (Jennifer Couper). Royal Melbourne Hospital, Vic (Peter Colman, John Wentworth, Leonard Harrison). Barwon Health, Vic (Peter Vuillermin). Monash Health, Vic (Georgia Soldatos). Children's Hospital at Westmead, NSW (Maria Craig). Royal Hospital for Women, NSW (Maria Craig). St George Hospital, NSW (Maria Craig). Princess Margaret Hospital/Perth Children's Hospital, WA (Elizabeth Davis, Aveni Haynes). Mater Mother's Hospital/Queensland Children's Hospital, QLD (Tony Huynh)

Lead Academic Sites: The University of Adelaide/Robinson Research Institute, SA (Jennifer Couper, Megan Penno, Rebecca Thomson, Kelly McGorm, Helena Oakey, Simon Barry). WEHI, Vic (Leonard Harrison, John Wentworth). University of New South Wales, NSW (Maria Craig, William Rawlinson). University of Sydney, NSW (Maria Craig). University of Western Australia/Telethon Kids Institute/Harry Perkins Institute, WA (Elizabeth Davis,

Aveni Haynes, Grant Morahan), University of Melbourne, Vic (Richard Sinnott). University of Queensland, QLD (Tony Huynh)

Satellite Recruitment/Birthing Sites (SA): Country Health SA (Jennifer Couper), Flinders Medical Centre (Brian Coppin), Lyell McEwin Hospital (Jennifer Couper), Ashford Hospital, Flinders Private Hospital, North Eastern Community Hospital.

Satellite Recruitment/Birthing Sites (VIC): Royal Women's Hospital (Alison Nankervis), Ballarat Base Hospital (David Song), Bendigo Health (Mark Savage), Epworth Geelong Hospital (Peter Vuillermin), Mercy Hospital for Women (Christine Houlihan, Peter Colman), St. John of God Geelong (Peter Vuillermin), Sunshine Hospital/ Joan Kirner Women's and Children's Hospital (Balasubramanian Krishnamurthy), Werribee Mercy Hospital (Sheetal Tipnis)

Satellite Recruitment/Birthing Sites (NSW): Hunter Diabetes Centre (Claire Morbey), John Hunter Hospital (Maria Craig), John Hunter Children's Hospital (Maria Craig), Sydney Children's Hospital (Maria Craig), The Sutherland Hospital (Maria Craig), Westmead Hospital (Maria Craig), North Shore Private Hospital (Maria Craig)

Satellite Recruitment/Birthing Sites (WA, all under Elizabeth Davis): Armidale Hospital, Bentley Hospital, Joondalup Health Campus, King Edward Memorial Hospital, Rockingham General Hospital, St John of God Mt. Lawley, St John of God Murdoch, St John of God Subiaco.

Satellite Recruitment/Birthing Sites (QLD): Royal Brisbane and Women's Hospital (Michael d'Emden), Wesley Hospital (Stephen Cook, Andrew Cotterill)

Satellite Recruitment/Birthing Sites (NT): Royal Darwin Hospital (Louise Maple-Brown)

Referring Physicians in Private: Natalie Harrison/Geelong Diabetes & Endocrinology (Vic), Dorothy Graham (WA), Linda McKendrick (SA), Amanda Terry (SA).

Other Birthing Hospitals: Albury-Wodonga Hospital, Angliss Hospital, Auburn Public Hospital, Bankstown Hospital, Bathurst Base Hospital, Beaudesert Hospital, Beijing United Family Hospital (China), Belmont Hospital, Berri Hospital, Blacktown Hospital, Box Hill Hospital, Broome Hospital, Buderim Private Hospital, Bunbury Hospital, Burnside War Memorial Hospital, Caboolture Hospital, Cabrini Hospital Malvern, Cairns Hospital, Cairns Private Hospital, Calvary Hospital, Calvary Hospital Bruce, Calvary Hospital Lenah Valley, Calvary Hospital Wagga, Calvary John James Hospital, Campbelltown Hospital, Canberra Hospital, Casey Hospital, Centenary Hospital for Women and Children, Coffs Harbour Base Hospital, Dandenong Hospital, Darwin Birth Centre, Darwin Private Hospital, Dubbo Base Hospital, Echuca Regional Health, Emerald Hospital, Epworth Freemasons Hospital, Fiona Stanley Hospital, Frances Perry House, Frankston Hospital, Gawler Hospital, Gippsland Health, Glengarry Private Hospital, Gold Coast Private Hospital, Gold Coast University Hospital, Gosford Hospital, Gosford Private Hospital, Goulburn Valley Health, Grafton Base Hospital, Greenslopes Private Hospital, Griffith Base Hospital, Gunnedah Hospital, Hawkes Bay Hospital (New Zealand), Hervey Bay Hospital, Hobart Private Hospital, Hornsby Hospital, Hurstville Private Hospital, Ipswich Hospital, Jessie McPherson Private Hospital, John Flynn Private Hospital, Kalgoorlie Hospital, Kapunda Hospital, Kareena Private Hospital,

Katoomba Hospital, Launceston General Hospital, Lismore Base Hospital, Liverpool Hospital, Logan Hospital, Mackay Base Hospital, Maitland Hospital, Manly Hospital, Mater Hospital Sydney, Mater Mothers' Hospital, Mater Mothers' Private Hospital, Mater Private Hospital Mackay, Mater Private Hospital Redland, Mater Women's and Children's Hospital Townsville, Mitcham Private Hospital, Moruya District Hospital, Mount Barker Hospital, Narrabri Hospital, Nepean Hospital, Nepean Private Hospital, Newcastle Private Hospital, North Gosford Private Hospital, North West Hospital, North West Private Hospital, Northeast Health Wangaratta, Northern Beaches Hospital, Northern Hospital, Northpark Private Hospital, Norwest Private Hospital, Orange Base Hospital, Osborne Park Hospital, Peel Health Campus, Peninsula Private Hospital, Pindara Private Hospital, Port Augusta Hospital, Port Macquarie Hospital, Prince of Wales Hospital, Prince of Wales Private Hospital, Queanbeyan District Hospital, Queen Victoria Hospital, Redcliffe Hospital, Redland Hospital, Riverland General Hospital, Rockhampton Base Hospital, Royal Hobart Hospital, Royal Hospital for Women, Royal North Shore Hospital, Royal Prince Alfred Hospital, Sandringham Hospital, St George Private Hospital, St John of God Ballarat Hospital, St John of God Bendigo Hospital, St John of God Berwick Hospital, St John of God Bunbury Hospital, St John of God Midland Hospital, St Vincent's Hospital, St Vincent's Private Hospital, St Vincent’s Private Hospital Toowoomba, Stanthorpe Hospital, Sunnybank Private Hospital, Sunshine Coast University Hospital, Swan District Hospital, Sydney Adventist Hospital, Tamworth Hospital, Tanunda Hospital, The Canberra Hospital, The Tweed Hospital, Toowoomba Base Hospital, Toowoomba Private Hospital, Townsville University Hospital, Wallaroo Hospital, Wangaratta Hospital, Waverley Private Hospital, Westmead Private Hospital, Wodonga Hospital, Wollongong Hospital, Wollongong Private Hospital.

We also gratefully acknowledge the participants and their families who contribute to the ENDIA Study.
